# Epigenetic dynamics during capacitation of naïve human pluripotent stem cells

**DOI:** 10.1126/sciadv.adg1936

**Published:** 2023-09-29

**Authors:** João Agostinho de Sousa, Chee-Wai Wong, Ilona Dunkel, Thomas Owens, Philipp Voigt, Adam Hodgson, Duncan Baker, Edda G. Schulz, Wolf Reik, Austin Smith, Maria Rostovskaya, Ferdinand von Meyenn

**Affiliations:** ^1^ Laboratory of Nutrition and Metabolic Epigenetics, Department of Health Sciences and Technology, ETH Zurich, 8603 Schwerzenbach, Switzerland.; ^2^ Systems Epigenetics, Otto Warburg Laboratories, Max Planck Institute for Molecular Genetics, 14195 Berlin, Germany.; ^3^ Epigenetics Programme, The Babraham Institute, Cambridge CB22 3AT, UK.; ^4^ School of Biosciences, The Julia Garnham Centre, University of Sheffield, S10 2TN Sheffield, UK.; ^5^ Sheffield Diagnostic Genetics Services, Sheffield Children’s NHS Foundation Trust, S5 7AU Sheffield, UK.; ^6^ Wellcome Trust Sanger Institute, Hinxton, Cambridge CB10 1QR, UK.; ^7^ Cambridge Stem Cell Institute, University of Cambridge, Cambridge CB2 0AW, UK.; ^8^ Centre for Trophoblast Research, University of Cambridge, Cambridge CB2 3EG, UK.; ^9^ Altos Labs Cambridge Institute of Science, Cambridge CB21 6GP, UK.; ^10^ Living Systems Institute, University of Exeter, EX4 4QD Exeter, UK.; ^11^ Department of Medical and Molecular Genetics, King’s College London, Guy’s Hospital, SE1 9RT London, UK.

## Abstract

Human pluripotent stem cells (hPSCs) are of fundamental relevance in regenerative medicine. Naïve hPSCs hold promise to overcome some of the limitations of conventional (primed) hPSCs, including recurrent epigenetic anomalies. Naïve-to-primed transition (capacitation) follows transcriptional dynamics of human embryonic epiblast and is necessary for somatic differentiation from naïve hPSCs. We found that capacitated hPSCs are transcriptionally closer to postimplantation epiblast than conventional hPSCs. This prompted us to comprehensively study epigenetic and related transcriptional changes during capacitation. Our results show that CpG islands, gene regulatory elements, and retrotransposons are hotspots of epigenetic dynamics during capacitation and indicate possible distinct roles of specific epigenetic modifications in gene expression control between naïve and primed hPSCs. Unexpectedly, PRC2 activity appeared to be dispensable for the capacitation. We find that capacitated hPSCs acquire an epigenetic state similar to conventional hPSCs. Significantly, however, the X chromosome erosion frequently observed in conventional female hPSCs is reversed by resetting and subsequent capacitation.

## Teaser

hPSCs capacitation in vitro recapitulates features of early human development and gives insights into epigenetic dynamics.

## INTRODUCTION

Pluripotency describes a dynamic cellular state, conferring the potential to develop into all embryonic lineages. Pre- and postimplantation epiblast cells are both pluripotent yet have distinct properties, including transcriptional, epigenetic, metabolic, and cell polarity (*1*–*5*). By using different culture conditions, pluripotent stem cells (PSCs) can be derived in the states corresponding to these two extremes of epiblast progression, termed naïve and primed PSCs, respectively (*6*, *7*). Naïve pluripotent cells are unresponsive to somatic lineage induction cues and only acquire the competence for differentiation following the transition to primed pluripotency (capacitation) (*8*–*10*).

Epigenetic modifications are the main means by which cellular identity is maintained during development and differentiation, ensuring unidirectional specialization and determination (*11*). Their cross-talk with chromatin modifiers, transcription, and metabolism is key to understanding how cells maintain their identity and respond to differentiation cues (*12*). Characterizing the molecular dynamics of pluripotency transition during human embryonic development has been challenging, and much of our current knowledge is derived from model organisms, such as the mouse, with a gestational period, metabolism, transcriptional characteristics, and genetic composition different from human (*13*–*16*). Recently, naïve and primed human PSCs (hPSCs) have been shown to have distinct epigenetic landscapes, with different levels and distribution of DNA methylation, H3K27me3, polycomb-repressive complexes (PRCs), and specific enhancer activity and interactions (*10*, *17*–*19*). However, studies of human pluripotency frequently use hPSCs that have been derived and long-term cultured in the primed state, also called conventional hPSCs, known to acquire culture-induced epigenetic aberrations in DNA methylation and X chromosome state (*20*, *20*–*23*), and therefore might incorrectly reproduce characteristics of postimplantation epiblast. We have established an hPSC-based in vitro model of human epiblast developmental transition, recapitulating its transcriptional properties and timing (*9*). In this study, we set out to characterize the epigenetic dynamics of human pluripotency with this capacitation system. We have generated global epigenetic maps of active and repressive histone modifications, chromatin accessibility, and DNA methylation from naïve, capacitated, and conventional hPSCs and correlated these with our previously reported gene expression datasets from the same system. The results provide a comprehensive analysis of the epigenetic dynamics between pluripotency states and reveal differences between capacitated and conventional hPSCs.

## RESULTS

### Capacitated hPSCs map transcriptionally closer to embryonic postimplantation epiblast than conventional hPSCs

**Fig. 1.**
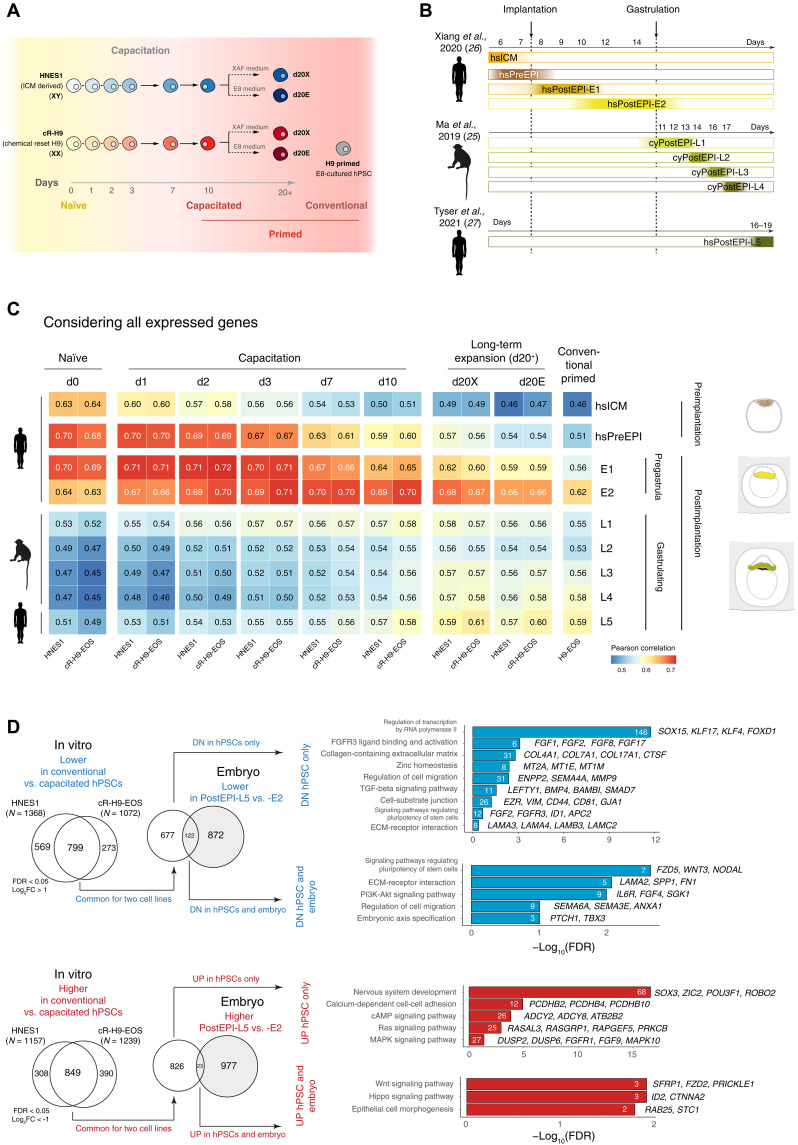
Mapping the pluripotency states to primate embryonic epiblast progression. (**A**) Experimental setup of RNA-seq during the time course of capacitation (*9*). (**B**) Single-cell RNA-seq data from embryos used for the comparison, aligned along developmental time (*25*–*27*). (**C**) Pearson correlations between hPSCs during capacitation in vitro and embryonic epiblast at different developmental stages, calculated using all expressed genes. (**D**) Comparison of gene sets that differ between capacitated and conventional hPSCs to genes dynamically expressed during the embryonic epiblast progression. A majority of genes differentially expressed between capacitated and conventional hPSCs did not match their dynamics in the embryonic epiblast (left-hand side). Gene Ontology of gene sets that match and do not match between in vitro and in vivo systems (right-hand side). FDR, false discovery rate; FC, fold change; ECM, extracellular matrix; hs, human; cy, cynomolgus monkey; ICM, inner cell mass; PreEPI, preimplantation epiblast; PostEPI, postimplantation epiblast; UP, upregulated; DN, downregulated.

Our previous work showed that capacitation in vitro occurs during 10 days of culturing the naïve hPSCs in the presence of WNT inhibitors, and it follows human and nonhuman primate embryonic epiblast transcriptional dynamics (*9*, *24*). Yet, the exact staging of hPSCs during this process was not feasible because the transcriptomic data from embryos did not extend beyond the onset of gastrulation. Recently, an extended characterization of primate embryos has become available, providing additional resources for mapping hPSCs during capacitation to the stages of embryonic epiblast development (*25*–*27*). We used our published transcriptome profiling of two hPSC lines, an embryo-derived HNES1 and a chemically reset cR-H9-EOS, during capacitation, as well as following additional longer-term expansion in two media [XAF containing tankyrase inhibitor XAV939, Activin A, and fibroblast growth factor (FGF2); as well as commercial E8] and the parental conventional primed hPSCs H9-EOS used for resetting [GSE123055; (*9*)]. The following publicly available single-cell RNA sequencing (RNA-seq) datasets were integrated for the comparison: in vitro–cultured human embryos from preimplantation to pregastrulation stage, in vitro–cultured gastrulating macaque embryos, and in utero human gastrula (Fig. 1, A and B, and tables S1 and S2) (*25*–*27*).

We found a notable change in the transcriptional properties of embryonic epiblast at the onset of gastrulation, which was neither associated with species (human versus nonhuman) nor with embryo source (in vitro versus in utero) (fig. S1A). We then conducted a Pearson correlation analysis using all expressed genes (Fig. 1C) and the most differentially expressed genes in hPSCs (fig. S1B and table S3) between our RNA-seq from hPSCs and the single-cell integrated RNA-seq dataset from embryos. Consistent with previous analyses, the results showed that naïve hPSCs were most similar to the preimplantation epiblast (Pearson correlations, 0.68 to 0.70 and 0.79, respectively). Furthermore, hPSCs progressively became similar to postimplantation stages during capacitation and were most similar to the pregastrulation epiblast after 10 days of capacitation (Pearson correlations, 0.69 to 0.70 in both analyses). During extended culture, capacitated hPSCs increased similarity to later gastrulating epiblast while remaining most similar to the pregastrulating stage globally (Pearson correlations, 0.66–0.68) (Fig. 1C). Conventional H9-EOS hPSCs that had been derived and maintained long-term in primed conditions showed similarity to both pre- and gastrulating epiblasts; however, this similarity was lower compared to all other hPSC populations in the dataset (Pearson correlations, 0.62 and 0.59, respectively).

We then asked whether transcriptional differences between capacitated and conventional primed hPSCs resemble the dynamics of embryonic epiblast during progression to gastrulation or represent an artifact of in vitro culturing. We identified 799 genes with lower expression and 849 genes with higher expression in conventional primed hPSCs as compared to capacitated cells. Only a small fraction of these genes matched the changes in human embryos (122 and 23 genes, respectively; Fig. 1D), indicating that long-term cultured conventional primed hPSCs likely accumulate transcriptional changes not related to developmental progression. Gene Ontology (GO) analysis (table S4) showed that the few genes matching in vivo dynamics included members of the Wnt, Hippo, and phosphatidylinositol 3-kinase signaling pathways. In contrast, in vitro–specific down-regulated genes include a large group of transcription factors; members of the transforming growth factor–β pathway; and molecules involved in cell adhesion, migration, and extracellular matrix production, while in vitro–specific up-regulated genes were associated with mitogen-activated protein kinase, Ras, and cyclic adenosine 3′,5′-monophosphate signaling and neural development. Overall, we find that capacitated hPSCs appear transcriptionally closer to embryonic postimplantation epiblast than long-term cultured conventional primed hPSCs, which show transcriptional differences not related to in vivo developmental progression.

### Capacitated hPSCs have a similar epigenetic landscape to conventional primed hPSCs

Some epigenetic differences between naïve and conventional primed hPSCs were highlighted in previous studies (*10*, *17*, *18*, *28*, *29*). However, the epigenetic changes in cells that transitioned in vitro from naïve to primed have not been thoroughly characterized. Moreover, our transcriptomic analysis revealed that capacitated hPSCs better match the postimplantation epiblast than the conventional cells (Fig. 1). Hence, aiming to characterize the epigenetic landscape of naïve and primed pluripotent cells and to identify potential epigenetic differences between capacitated and conventional primed hPSCs, we performed a global chromatin profiling of naïve, capacitated, and conventional primed hPSCs.

**Fig. 2.**
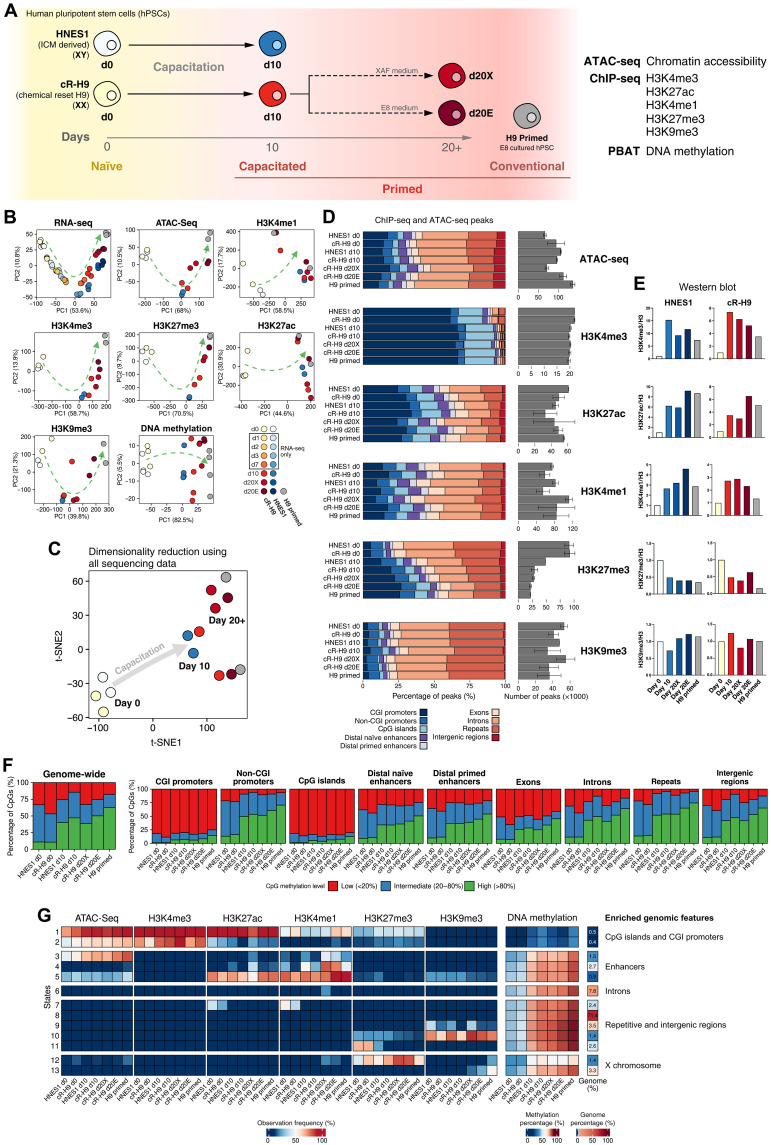
Epigenetic profiling of human naïve and primed PSCs. (**A**) Diagram of the experimental design for the ATAC-seq, ChIP-seq, and PBAT (Post-bisulfite adaptor tagging) sequencing assays. (**B**) Principal component analysis of the top 500 most variable differentially expressed genes, histone modifications ChIP-seq differential peaks, ATAC-seq differential peaks, and DNA methylation average in 200 CpG-containing genomic windows between all conditions. (**C**) t-distributed stochastic neighbor embedding (t-SNE) plot generated with the same data input as the PCA and with the R package MOFA2 used to integrate the datasets. (**D**) Percentage of ChIP-seq (H3K4me3, H3K27me3, H3K4me1, H3K27me3, and H3K9me3 marks) and ATAC-seq peaks overlapping annotated genomic regions and the total number of peaks for each condition. (**E**) Western blot normalized results for each histone modification and condition. (**F**) Genome-wide DNA methylation percentage distribution calculated at a CpG resolution and DNA methylation percentage distribution over annotated genomic regions at a CpG resolution. The CpG methylation levels were divided into three categories: In red, “Low,” with a methylation percentage below 20%; in blue, “Intermediate,” with a methylation percentage between 20 and 80%; and in green, “High,” with a methylation percentage above 80%. (**G**) ChromHMM 13-state genome-wide model built using ATAC-seq and histone modifications’ ChIP-seq aligned reads. The heatmap shows the observation frequency of histone modifications and chromatin accessibility across different conditions and includes the average methylation percentage, the percentage of the genome classified with each state, and the top enriched regions. Only autosomes were considered for all analyses of this figure, except for ChromHMM, which included the X and Y chromosomes. CGI, CpG island.

We profiled the histone modifications H3K4me3, H3K4me1, H3K27me3, H3K27ac, and H3K9me3 using ChIP-seq (chromatin immunoprecipitation followed by sequencing), chromatin accessibility using ATAC-seq (assay for transposase-accessible chromatin with sequencing), and DNA methylation using PBAT (post-bisulfite adaptor tagging). We characterized all conditions using genetically matched cells: conventional primed H9-EOS hPSCs; chemically reset naïve cells cR-H9-EOS, derived from the H9-EOS cell line; capacitated cR-H9-EOS after 10 days of capacitation; and capacitated cR-H9-EOS followed by additional expansion in XAF or E8 medium. In addition, we profiled embryo-derived HNES1 in the naïve state and after 10 days of capacitation (Fig. 2A and table S5). To determine whether capacitation was selected for chromosome abnormalities, we performed a cytogenetic analysis of the capacitated cells. The results revealed that the cR-H9-EOS cells capacitated and subsequentially expanded for 20 and 50 days in two independent experiments were karyotypically intact (46XX; 26 of 30 and 29 of 30 metaphases) (fig. S2, A to C).

Using a principal components analysis (PCA) and hierarchical clustering, we observed that each epigenetic modification separated naïve from capacitated (d10, d20X, and d20E) cells, highlighting that epigenetic remodeling during the naïve-to-primed transition affects all modifications, irrespective of their activate or repressive role (Fig. 2B and fig. S3A). Furthermore, a dimensionality reduction analysis integrating all data types showed a clear “epigenetic” and transcriptional transition from naïve-to-primed states (Fig. 2C). However, we noticed that d10 capacitated cells were slightly separated from conventional primed hPSCs, highlighting their epigenetic distinction, with long-term expansion resulting in cells with a more similar epigenetic landscape to conventional primed cells. This difference was more evident in the chromatin accessibility, H3K4me3, and DNA methylation results (Fig. 2B and fig. S3A). Histone marks H3K27ac and H3K4me1, associated with distal regulatory elements, clustered the d10, d20X, and d20E capacitated cells together, suggesting that enhancers are remodeled during capacitation and remain relatively stable once the primed state is established (Fig. 2B and fig. S3A). In addition, culture conditions contributed to the epigenetic characteristics: E8-cultured conventional hPSCs and E8-expanded capacitated hPSCs formed closer clusters in most sequencing assays (Fig. 2, B and C). These culture condition differences were also highlighted in a genome-wide correlation analysis of 2-kb sized genomic regions where H3K9me3 showed a pronounced difference between hPSCs cultured in XAF and E8 medium, underscoring the effect of culture systems on the epigenetic landscape (fig. S3B). Furthermore, by performing a correlation analysis between epigenetic modifications in all conditions (fig. S3C), we observed a decreased correlation between DNA methylation and the remaining epigenetic modifications and chromatin accessibility levels in the long-term cultured cells compared to the naïve and capacitated cells.

We then characterized the genome-wide distribution of ChIP-seq peaks for the histone modifications and ATAC-seq for chromatin accessibility (Fig. 2D). In addition, we measured the global levels of modified histones by Western blot (Fig. 2E and fig. S4, A to D). The total amount of histone marks assessed by Western blot showed a substantial increase in H3K4me3, H3K4me1, and H3K27ac levels despite their relative distribution across genomic features remaining largely unaffected between pluripotency states. In contrast, the number of H3K27me3 ChIP-seq peaks substantially decreased during capacitation, with proportion of peaks overlapping CpG islands increasing, indicating redistribution of H3K27me3 (Fig. 2D). Western blot showed a global decrease in H3K27me3 levels, in agreement with previously published data (*19*), indicating that H3K27me3 is redistributed to occupy fewer regions in primed cells and mainly to focus on CpG islands. All these characteristics were shared between capacitated (d10, d20X, and d20E) and conventional primed cells.

DNA methylation increased globally during the transition from naïve-to-primed state (Fig. 2F and fig. S4, E and F), in agreement with previous observations (*10*, *28*). This increase affected most genomic regions, including naïve- and primed-specific enhancers, non-CpG island–containing promoters, genic and intergenic regions, and repeats, but not CpG islands. However, previous studies also showed that naïve hPSCs fail to maintain the allele-specific methylation status of imprinting control regions (ICRs) (*10*, *29*). We confirmed this observation and found that capacitation does not restore allele-specific DNA methylation in those regions (fig. S4G and table S7). Analysis of histone modifications and DNA methylation in these ICRs showed that allele-specific chromatin accessibility, H3K4me3, and H3K9me3 were also lost in most ICRs during resetting and were not restored.

A hidden Markov model (HMM) analysis segmented the genome into 13 chromatin states based on their specific combination of histone modification and accessibility across all samples (Fig. 2G and table S6). States 1 and 2 showed low DNA methylation level and represented CpG-associated regions with active and inactive promoter signatures, respectively. Other chromatin states increased DNA methylation levels during capacitation and included enhancers (states 3 to 5), introns (state 6), repetitive regions (states 7 to 11, including those gaining H3K9me3 or losing H3K27me3), and X chromosome–associated regions (state 12, which displayed the expected gain of H3K27me3 in female cR-H9 cells during capacitation but a remarkably reduced level in conventional H9 cells and state 13 with elevated K9me3 in the conventional cells).

In summary, our profiling revealed global epigenetic rewiring during capacitation. hPSCs after 10 days of capacitation become epigenetically similar to the conventional primed hPSCs, although with some differences. Capacitated hPSCs progressively become epigenetically closer to conventional primed hPSCs during extended culturing, being more similar with matching culture conditions.

### PRC2 inhibition does not interfere with pluripotency

We were intrigued by the global changes in the levels of epigenetic modifications during capacitation and asked whether they correlated with the levels of epigenetic modifiers. While multiple genes encoding epigenetic modifiers were dynamically expressed (such as *ASH2L*, *KMT2A*, *KMT2C*, *KDM6A*, and *DNMT3L*), as also reported by others (*19*), only a subset of subunits per complex were affected (fig. S5, A to C, and table S8). Thus, the expression levels of these modifiers may not sufficiently explain the global epigenetic changes during capacitation.

**Fig. 3.**
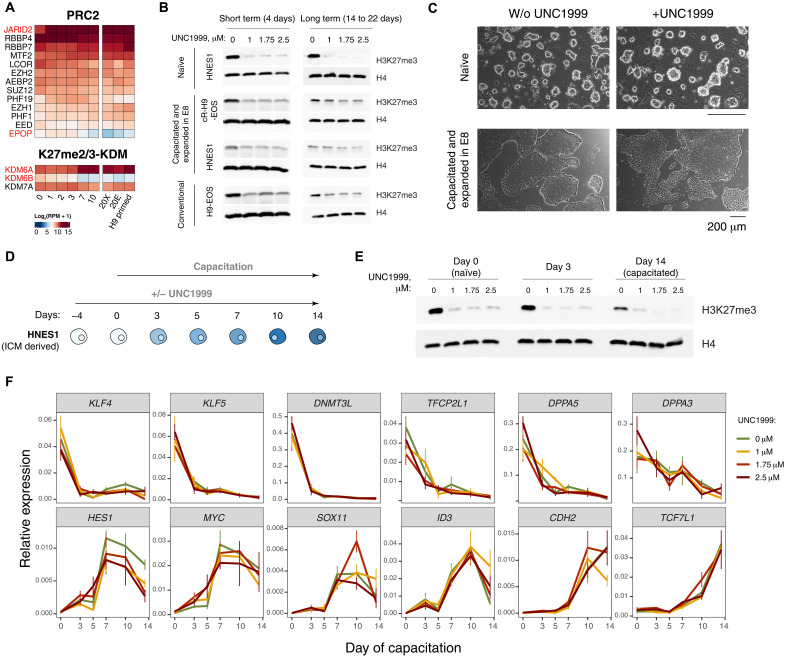
PRC2 inhibition does not interfere with the pluripotency compartment in vitro. (**A**) Expression of the members of H3K27me3 writing and erasing complexes during capacitation. (**B**) Inhibition of PRC2 activity with UNC1999 inhibitor reduces the global levels of H3K27me3 in hPSCs, shown by Western blot. (**C**) Morphology of naïve and primed hPSCs after extended culturing with PRC2 inhibitor UNC1999. (**D**) Experimental design of testing the effect of PRC2 inhibition during capacitation. (**E**) Reduced levels of H3K27me3 in hPSCs during capacitation in the presence of UNC1999, shown by Western blot. (**F**) Marker expression during capacitation in the presence of PRC2 inhibitor UNC1999, shown by quantitative real-time PCR (qRT-PCR). KDM, histone lysine demethylase; RPM, reads per million.

The global reduction of H3K27me3 was one of the most prominent changes during capacitation. Among the H3K27me3 writers (PRC2 complex) and erasers (*KDM6A*, *KDM6B*, and *KDM7A*), only a transcriptional increase in *KDM6A* correlated with H3K27me3 reduction (Fig. 3A). Because H3K27me3 reduction was prominent in repeat elements (Fig. 2D and state 11 from Fig. 2G), we tested whether H3K27me3 had a considerable role in silencing transposable elements in naïve hPSCs. We re-analyzed publicly available RNA-seq datasets (*19*, *30*, *31*) and observed no or only minor up regulation of transposable elements in naïve hPSCs treated with enhancer of zeste homolog 1 and 2 (EZH1/2) inhibitors (fig. S6, A and B, and table S9) or in EZH2-deficient primed hPSCs (fig. S6C and table S9).

We sought to establish the effect of H3K27me3 depletion in naïve, capacitated, conventional primed hPSCs and during the naïve-to-primed transition. Previous studies showed that acute inhibition of EZH1/2 activity does not interfere with naïve or primed pluripotency (*19*, *31*). Moreover, EZH1/2 inhibition facilitates differentiation of naïve hPSCs to extraembryonic lineages, while EZH2 depletion results in the derepression of developmental programs in conventional primed hPSCs (*30*). Hence, PRC2 was proposed to establish a roadblock between pluripotency and lineage commitment. Furthermore, because of a slight up-regulation of primed-specific genes in the naïve hPSCs treated with EZH2 inhibitor, H3K27me3 was also proposed to represent a roadblock between the pluripotency states (*31*), but this was never experimentally verified. We therefore treated naïve HNES1, capacitated HNES1 and cR-H9-EOS, and conventional primed H9-EOS with an EZH1/2 inhibitor (UNC1999). Our results confirmed a global H3K27me3 reduction after 4 or more days of treatment with the UNC1999 inhibitor at different concentrations (1, 1.75, and 2.5 μM) by Western blot (Fig. 3B). The inhibition did also not induce substantial changes in the expression of pluripotency markers in naïve (fig. S7A) or primed hPSCs (fig. S7B). Moreover, extended passaging in the presence of the UNC1999 inhibitor for 14 to 22 days did not change cell morphology (Fig. 3C) nor affect the expression of pluripotency factors (fig. S7, D and E).

Next, we acutely depleted H3K27me3 in naïve HNES1 and performed capacitation in the presence of UNC1999 (Fig. 3D). H3K27me3 remained considerably reduced throughout the capacitation (Fig. 3E). Unexpectedly, the dynamics of major naïve (*KLF4*, *KLF5*, *DNMT3L*, *TFCP2L1*, *DPPA3*, and *DPPA5*) and primed (*HES1*, *MYC*, *SOX11*, *ID3*, *CDH2*, and *TCF7L1*) pluripotency markers were not substantially changed during capacitation by PRC2 inhibition (Fig. 3F). Therefore, in contrast to existing propositions, H3K27me3 depletion neither facilitated nor prevented the pluripotent state transition.

### Genomic hotspots of epigenetic dynamics during pluripotency state transitions

**Fig. 4.**
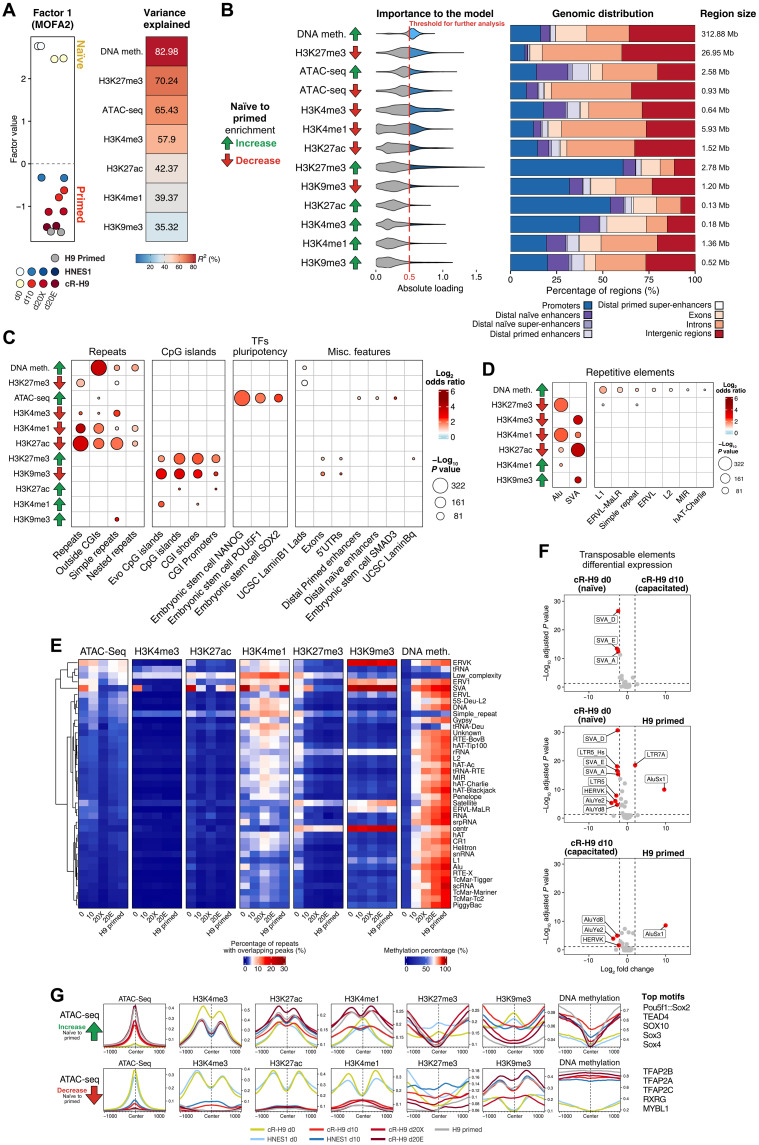
Modeling of epigenetic enrichment dynamics across pluripotency states. (**A**) MOFA2 factor values for factor 1 extracted from differential peaks of histone modifications’ ChIP-seq, ATAC-seq, and the top variable 200 CpG-containing regions based on methylation percentage between conditions. On the right side, the *R*^2^ values indicating the variance explained by each sequencing assay in the sample clustering of factor 1. (**B**) Top features ordered by their model loading weight (expressed as “importance to the model”). On the right side, the genomic distribution and size of the top features selected by having an absolute loading weight above 0.5 in factor 1. (**C**) Enrichment of the top features in factor 1 over annotated genomic regions. Only results with an FDR below 0.05 and a −log *P* value above 40 are shown. (**D**) Enrichment of the top features in factor 1 over repetitive elements. The repeat element names are given by the repeat family. Only results with an FDR below 0.05 and a −log *P* value above 40 are shown. (**E**) DNA methylation percentage and percentage of ATAC-seq and ChIP-seq peaks overlapping repeat elements. The repeat element names are given by the repeat family. (**F**) Differential expression of transposable element classes between “cR-H9 d0” and “cR-H9 d10” cells, “cR-H9 d10” and “H9 primed” cells, and “cR-H9 d10” and “H9 primed” cells. The results in red were selected on the basis of an absolute log_2_ fold change greater than 2 and an adjusted *P* value of less than 0.05. (**G**) Epigenetic profile of regions centered at the top ATAC-seq peaks, selected based on an absolute loading weight above 0.5 in factor 1. On the right side, the top results of the MEME Suite motif analysis. All results in this figure were limited to autosomes. 5′UTR, 5′ untranslated region.

After a genome-wide overview of the epigenetic dynamics, we sought to identify the genomic features with substantial epigenetic changes between pluripotency states. To this end, we performed a multiomics factor analysis, using the Bioconductor package Multi-Omics Factor Analysis 2 (MOFA2) (*32*), to identify the principal sources of variation from our multiomic and multisample datasets. This modeling approach extracts factors that represent the driving sources of variability across the data and allows us to estimate the relative contribution of different assays to the variation between the samples. In our analysis, using the most variable regions for each epigenetic modification, the model extracted one factor that separated the samples by their pluripotency state, factor 1. The sample clustering produced by this factor was mostly driven by a DNA methylation increase and H3K27me3 decrease, confirming that these are the most significant epigenetic changes during the naïve-to-primed transition, with changes in chromatin accessibility and H3K4me3 also having an important role (Fig. 4A and fig. S8A).

We then explored the genomic distribution of the epigenetic regions with the highest contribution to factor 1 and conducted an enrichment analysis over functional genomic features (Fig. 4, B and C). We observed that these regions significantly overlapped repetitive elements, CpG islands, and pluripotency associated transcription factor binding sites (Fig. 4C). In agreement with the results from the genome-wide epigenetic characterization (Fig. 2), a subset of regions with increasing DNA methylation and decreasing H3K27me3 levels between naïve and primed overlapped repetitive elements outside CpG islands. Unexpectedly, some regions with the highest contribution to factor 1 showing decreasing levels of H3K9me3 between naïve and primed had a strong enrichment in CpG islands and were located near (less than 50 kb) genes related to organ development (Fig. 4C and fig. S8, B and C). However, the CpG islands that overlapped these regions were shorter and more enriched in gene bodies than CpG islands gaining H3K27me3, which were long and mostly near transcription start sites (TSSs) (fig. S8B). Moreover, regions with increased levels of H3K4me1 in primed as compared to naïve hPSCs had a significant overlap with distal CpG islands, also known as “orphan” CpG islands (Fig. 4C and fig. S8B). The removal of H3K9me3, increase in H3K4me1, and gain of H3K27me3 in CpG islands may therefore reflect the regulatory role of these distal and proximal regions during induction of developmental genes (*33*), which is enabled during capacitation.

A more detailed analysis of repetitive elements revealed that some groups of retrotransposons had differential epigenetic marks between the two pluripotency states (Fig. 4, D and E). Most of those elements, which included L1, L2, and endogenous retrovirus-like (ERVL), gained DNA methylation and reduced H3K27me3 without major remodeling of other histone modifications during capacitation. Furthermore, we also observed distinct dynamics of histone modification in Alu and SVA elements between pluripotency states. In naïve hPSCs, Alu elements showed higher levels of poised enhancer-associated marks, H3K4me1 and H3K27me3, whereas SVA elements were highly accessible and overlapped by active promoter-associated marks, H3K4me3 and H3K27ac, which was consistent with their higher expression in naïve compared to primed hPSCs (Fig. 4F). We also noticed higher expression and accessibility of ERV1 and ERVK elements in the naïve hPSCs, which is in agreement with previous reports (*34*).

Our analysis also underscored that during capacitation, a subset of regulatory regions became more accessible and overlapped putative distal enhancers [from Barakat *et al.* (*35*)] (Fig. 4C). Intriguingly, these regions were enriched for primed-specific binding sites of NANOG, OCT4 (POU5F1), and SOX2, known as canonical pluripotency transcription factors, and GO analysis revealed an association with genes involved in organ morphogenesis (fig. S8C). During naïve-to-primed transition, these regions lost H3K27me3 and H3K9me3 marks and maintained low DNA methylation levels. Concomitantly, H3K27ac and H3K4me1 modifications changed their profile: They went from having the highest value at the center of the accessibility regions in the naïve state to flanking the peak region in the primed state (Fig. 4G and fig. S8, D and E). Motif analysis revealed that the POU5F1-SOX2 heterodimer motif, individual motifs for POU5F1 and SOX family, and TEAD4 putative binding sites were enriched in the primed-specific accessible regions. The binding motifs for naïve pluripotency factor transcription factor AP-2 gamma (TFAP2C (*36*) and major regulators of extraembryonic fates TFAP2A and TFAP2B were enriched in naïve-specific accessible regions (Fig. 4G, fig. S8F, and table S10).

In summary, the transition from naïve-to-primed pluripotency in hPSCs is characterized epigenetically by global DNA methylation and H3K27me3 changes, with regulatory elements (including CpG islands) and repetitive elements being hotspots for those changes. In addition, we observed epigenetic and accessibility remodeling in regulatory regions that were associated with pluripotency factor binding.

### Global association between gene expression and promoter epigenetic modifications dynamics

**Fig. 5.**
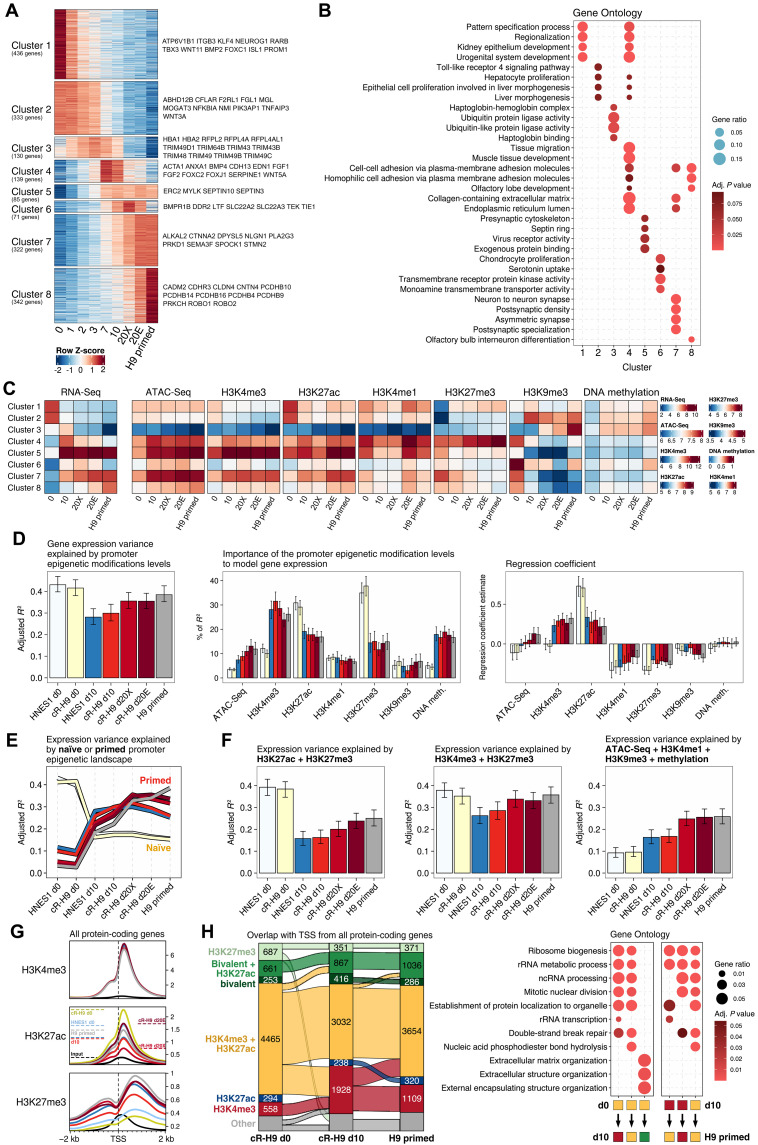
Association between promoter epigenetic state and gene expression. (**A**) RNA-seq gene clusters defined based on their significant expression differences between all conditions and expression pattern. (**B**) Top Gene Ontology terms associated with each RNA-seq cluster. (**C**) Average expression of the RNA-seq clusters genes with their average promoter chromatin accessibility and epigenetic modifications normalized counts. (**D**) Multivariate linear regression using gene expression as the target variable and the levels of promoter epigenetic modifications as predictor variables. Only the RNA-seq cluster genes were selected for this regression analysis. On the right side, the relative importance of each variable in the multivariate linear regression model measured by the “lmg” metric from the R package relaimpo and the regression coefficient for each variable and condition of the multivariate linear regression model. (**E**) Gene expression variance explained by the promoter epigenetic modification levels from other conditions. (**F**) Multivariate linear regression modeling gene expression based on promoter H3K27ac and H3K27me3 levels; promoter H3K4me3 and H3K27me3 levels; and promoter ATAC-seq, H3K4me3, H3K9me3, and methylation levels. (**G**) Read profiles from H3K4me3, H3K27ac, and H3K27me3 histone modification overlapping the TSS ± 2 kb in all protein-coding genes. (**H**) Number of protein-coding genes with their TSS categorized based on having an overlap with the following combination of ChIP-seq peaks in H9 cells: only H3K27me3, “H3K27me3”; H3K4me3 with H3K27me3 and H3K27ac, “Bivalent + H3K27ac”; H3K4me3 with H3K27me3, “Bivalent”; H3K4me3 with H3K27ac, “H3K4me3 + H3K27ac”; only H3K27ac, “H3K27ac”; only H3K4me3, “H3K4me3”; and the category “Other” containing all the remaining combinations between the epigenetic marks H3K4me3, H3K27ac, and H3K27me3. Only results from cR-H9 day 0, cR-H9 day 10, and H9 primed cells are shown. On the right side, the Gene Ontology from selected category transitions between conditions. All results in this figure were limited to autosomes. rRNA, ribosomal RNA; ncRNA, noncoding RNA.

Aiming to explore how epigenetic modifications associate with gene expression, we analyzed the epigenetic dynamics at promoters of differentially expressed genes in hPSCs (Fig. 5A and table S11). To this end, we first defined eight clusters from differentially expressed genes between all samples in the RNA-seq dataset. This clustering analysis separated the genes into two main groups with different dynamics during capacitation: downregulated genes (clusters 1 and 2) and upregulated genes (clusters 3 to 8).

From our results, only a fraction of genes up-regulated during capacitation maintained their expression during extended culture in d20+ (in either XAF or E8) and conventional primed H9-EOS cells (cluster 5; 85 genes). A large proportion of genes had highly dynamic expression between these conditions (clusters 3 to 4 and 6 to 8; 1004 genes), consistent with our previous supervised analysis (Fig. 1D). Genes whose expression increased during capacitation and subsequently down-regulated upon extended culture (clusters 3 and 4; 269 genes) were enriched for genes involved in migration, adhesion and matrix production, epithelial tissues, and ubiquitin ligase activity (Fig. 5B and table S12). Genes with high expression in E8-cultured cells, d20E and conventional primed hPSCs (cluster 7; 322 genes), were enriched in GO terms associated with neural development, suggesting a possible expression bias toward this lineage in long-term cultured hPSCs due to culture conditions, potentially affecting the outcome when used in clinical applications (*15*). Last, a group of up-regulated genes in the conventional primed hPSCs relative to capacitated cells (cluster 8; 342 genes) were associated with cell-cell adhesion molecules.

We then analyzed the dynamics of epigenetic modifications in promoters of the differentially expressed gene clusters (Fig. 5C). Promoters of down-regulated genes showed a decreased level of active expression-associated marks, H3K4me3 and H3K27ac, and increased repressive marks, either H3K27me3 (cluster 1) or H3K9me3 and DNA methylation (cluster 2). The expression dynamics of genes up-regulated during capacitation were reflected in the increase in chromatin accessibility, H3K4me3 and H3K27ac, and reduction of H3K27me3 and H3K9me3 in their promoters, while DNA methylation remained relatively low in these regions.

Last, we also analyzed the gene expression and dynamics of epigenetic modifications in promoters from a subset of genes known to be associated with naïve pluripotency, general pluripotency, postimplantation epiblast, and lineage-specific expression (fig. S9A). We included to the analysis genes with bivalent promoters. Our results showed that the selected subset of lineage-specific and postimplantation epiblast genes had promoters with higher CpG density than all protein-coding genes at the level comparable to bivalent genes (fig. S9, A and B, and table S13), substantiating the connection between developmental genes and high CpG density promoters (*37*, *38*). In addition, the selected lineage-specific genes had a consistent promoter bivalent chromatin in all conditions (fig. S9A), although we cannot rule out that this is due to cell heterogeneity. While these results suggest function specific epigenetic regulation, further studies will be necessary to draw more definitive conclusions.

Our results showed that epigenetic modifications in promoters of dynamically expressed genes are overall in agreement with known correlations between epigenetic modifications and transcription and indicate that the expression levels of lineage-specific genes may depend on the resolution of their promoter bivalent status (*39*).

### The associations between epigenome and transcriptome shifts during capacitation

Our results revealed an association between changes in epigenetic modifications in promoters and the expression of the correspondent genes, suggesting a regulatory role in transcription. Previous studies showed that promoter histone modification levels could partially explain the gene expression variance in a multivariable linear model. Here, we applied a similar method to the differentially expressed genes (Fig. 5A) to determine promoter features associated with expression dynamics between naïve and primed pluripotency. To this end, we used promoter quantifications of histone modifications, DNA methylation, and chromatin accessibility to generate a model of gene expression in each condition for the differentially expressed genes. To assess our modeling results, we used adjusted *R*^2^, a measure of the goodness of fit of a model, that determines how much the gene expression variance can be explained by the epigenetic modification (and accessibility) levels in the respective gene promoters. In addition, we included a metric measuring the importance of each epigenetic modification (lmg metric; see Materials and Methods) and the regression coefficient, which describes the relationship between a predictor variable (i.e., each epigenetic modification in the gene promoter) and the response (i.e., the respective gene expression).

Naïve hPSCs had the highest *R*^2^ values (~40%), d10 capacitated cells had the lowest values (~30%), and d20+ capacitated and conventional primed hPSCs had an *R*^2^ of ~35%. A closer look at the model statistics showed that the importance of promoter epigenetic modification to model gene expression was remarkably different between naïve and primed cells. The modeling of the differential gene expression was mostly associated with promoter H3K27ac and H3K27me3 levels in the naïve hPSCs, whereas H3K4me3 had the highest relative importance in the primed cells. The remaining epigenetic modifications had relatively lower relevance to model gene expression in these states (Fig. 5D). We also found that the naïve promoter epigenetic landscape can better model primed gene expression (*R*^2^ of ~15 to 20%) than the primed promoter epigenetic landscape can model naïve gene expression (*R*^2^ of ~5 to 10%) (Fig. 5E).

Because we observed the relative importance of the H3K27ac and H3K27me3 promoter levels in modeling gene expression in naïve cells, we decided to test the same modeling approach with only a subset of epigenetic modifications as independent variables (Fig. 5F). Our goal was to reveal their importance to the model by excluding some of the epigenetic modification. A model with only H3K27ac and H3K27me3 promoter levels had the highest *R*^2^ difference between naïve and primed cells (~40% for naïve cells, ~15% for d10 cells, and 20 to 25% for d20+ and conventional primed cells). Furthermore, to assess the relevance of the epigenetic modifications in modeling gene expression in primed cells, a model using accessibility, DNA methylation, H3K4me1, and H3K9me3 promoter levels had the highest *R*^2^ in primed cells (~10% for naïve cells, ~15% for d10 cells, and 25% for d20+ and conventional primed cells). However, a model with only H3K4me3 and H3K27me3 promoter levels showed that naïve and extended culture primed cells have a similar *R*^2^, with d10 capacitated cells being lower than those two cell categories (~35% for naïve cells, d20+, and conventional primed cells and ~25% for d10 cells). Thus, although the complete model (Fig. 5D) is more accurate, taking into account the influence of all epigenetic modification levels in promoters, the results from the smaller models still suggest that H3K27ac has a more specific association with naïve expression of the differential genes compared to primed state expression.

We then explored whether naïve and primed cells have different combinations of epigenetic modifications in promoters of protein-coding genes. Here, we restricted the analysis to H3K27ac, H3K4me3, and H3K27me3 because those had the highest relative importance for modeling gene expression in naïve and primed cells. We observed that H3K27ac and H3K4me3 peaks mostly co-occur on promoters of protein-coding genes and rarely alone. By quantifying the ChIP-seq–normalized reads overlapping the TSS (Fig. 5G) and by classifying the genes based on enrichment peaks overlapping the TSS (Fig. 5H), a proportion of those genes (~1200 genes) lost H3K27ac marks during capacitation (fig. S10, A to D, and table S14). Those genes were associated with noncoding RNA (ncRNA) processing, ribosome biogenesis, and ribosomal RNA metabolic processes, although genes that maintained both H3K27ac and H3K4me3 marks close to their TSS had a similar functional enrichment. A smaller proportion of the genes with both H3K27ac and H3K4me3 marks in naïve cells gained H3K27me3 marks close to their TSS during capacitation, a characteristic of bivalent promoters. Overall, we observed an increase in the number of genes with bivalent promoters during capacitation. We observed similar results when considering the epigenetic marks overlapping TSS ± 2 kb (fig. S10E).

Collectively, this suggests that the roles of specific epigenetic modifications in gene expression control are possibly distinct between the naïve and the primed hPSCs. Moreover, another important observation was that the global relationships of epigenetic states and gene expression levels were matching between the capacitated and conventional primed hPSCs.

### Reversion of X chromosome erosion by a round of resetting and capacitation

Human preimplantation female epiblast cells have two transcriptionally active X chromosomes and compensate for the dosage of X-linked genes by dampening the transcription of both X chromosomes (*40*–*42*), features that are also recapitulated in naïve hPSCs (*10*, *43*, *44*). During implantation, one of the X chromosomes is randomly inactivated and remains inactive in somatic lineages (*45*). X chromosome inactivation is regulated by the long ncRNAs *XIST* and *XACT* and histone modifications: Active X chromosomes are bound by *XACT* with or without *XIST*, whereas the inactive X chromosome is coated by *XIST* and exhibit high H3K27me3 levels (*44*). Reflecting postimplantation development, one of the X chromosomes is inactivated in female primed hPSCs. However, in long-term cultured cells, including conventional hPSCs, the inactive X chromosome often partially regains transcriptional activity associated with the loss of *XIST* expression and H3K27me3-enriched domains and accumulation of H3K9me3 (*20*). This phenomenon is termed “erosion.”

**Fig. 6.**
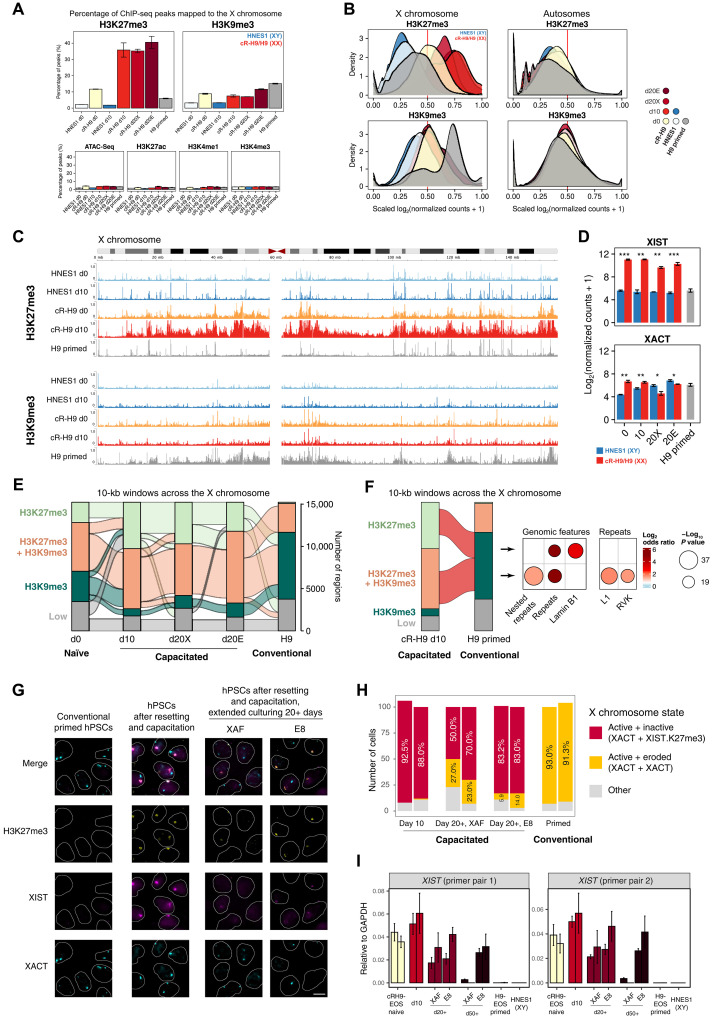
A round of resetting and capacitation of conventional hPSCs rescues X chromosome inactivation from an eroded landscape. (**A**) Percentage of all ChIP-seq and ATAC-seq peaks mapped to the X chromosome. (**B**) Distribution of scaled log-transformed normalized counts of H3K27me3 and H3K9me3 in 10-kb windows across the X chromosome and autosomes. The red line indicates the threshold used to categorize genomic regions as high or low H3K27me3 and H3K9me3. (**C**) H3K27me3 and H3K9me3 read count profiles across the X chromosome for naïve, capacitated day 10, and conventional H9 cells. (**D**) Gene expression levels of the long ncRNAs *XIST* and *XACT*. *P < 0.05, **P < 0.01, and ***P < 0.001, Student’s paired *t* test. Error bars indicate 1 SD of the mean values from three independent experiments. (**E**) Flow chart illustrating the number of 10-kb windows in the X chromosome classified based on H3K27me3 and H3K9me3 scaled log_2_-normalized counts threshold of 0.5. The classification includes windows containing high levels of H3K27me3 marks (H3K27me3), high levels of H3K9me3 marks (“H3K9me3”), high levels of both H3K27me3 and H3K9me3 marks (“H3K27me3 + H3K9me3”), and low levels of both marks (Low). (**F**) Flow chart illustrating the number of 10-kb windows in the X chromosome in the cR-H9 day 10 (capacitated) and H9 primed cells (conventional). On the right side is the enrichment analysis for genomic features and repeats of the regions represented in red. Only results with an FDR below 0.05, absolute odds ratio value above 2, and a −log *P* value above 10 are shown. (**G**) Immuno-FISH for H3K27me3, *XIST* and *XACT* in conventional primed hPSCs, cells after a round of resetting and capacitation, and cells after additional expansion in either XAF or E8. (**H**) Quantification of the immuno-FISH. Scale bar, 10 μm. (**I**) qRT-PCR for *XIST*. *GAPDH*, glyceraldehyde-3-phosphate dehydrogenase.

In our genome-wide characterization of the epigenetic landscape of naïve and primed pluripotency, the HMM identified two epigenetic states associated with the X chromosome: state 12, showing an increased H3K27me3 observation frequency, and state 13, showing an increase in DNA methylation during capacitation (Fig. 2G). Unexpectedly, state 12 regions had reduced H3K27me3 frequency in conventional primed hPSCs relative to capacitated cells, with both states showing higher H3K9me3 frequency in the conventional primed H9-EOS cells, suggesting X chromosome erosion. Upon examining the genome-wide distribution of ChIP-seq peaks, we confirmed that the percentage of H3K27me3 peaks mapped to the X chromosome in the conventional primed H9-EOS female cells was low and comparable to the male HNES1 cells. Concurrently, the percentage of H3K9me3 peaks mapped to the X chromosome was higher in conventional primed H9-EOS compared to the remaining cells (Fig. 6, A to C). These differences were observed only in X chromosome, not in autosomes, further confirming X chromosome erosion. Unexpectedly, the results also showed that after resetting and capacitation of the conventional primed H9-EOS, the percentage of peaks mapped to X chromosome increased for H3K27me3 and decreased for H3K9me3, suggesting that the eroded X chromosome reverted to an epigenetic landscape associated with inactivation during capacitation.

Analysis of *XIST* levels in the RNA-seq data further supported this hypothesis: *XIST* expression was lower in the female conventional primed H9-EOS cells comparable to the male HNES1 but was up-regulated following a round of resetting and maintained the same level of expression during and after capacitation (Fig. 6D). *XACT* expression did not considerably change during the resetting and capacitation round, except for d20X cells where *XACT* expression was reduced.

Next, we examined the dynamics of H3K27me3 and H3K9me3 marks in individual regions in the X chromosome. To this end, we binned the X chromosome into 10-kb windows and classified them by the H3K27me3 and H3K9me3 levels in each condition and tracked their states (Fig. 6E and fig. S11, A to C). In agreement with the number of peaks of these histone modifications and their density (Fig. 6, A and B), the conventional H9-EOS cells had only a minor fraction of H3K27me3-only marked regions, a reduced number of H3K27me3/H3K9me3 regions, and a substantial proportion of H3K9me3-only regions as compared to the naïve and capacitated cells. In addition, H3K27me3 was stably maintained on the X chromosome in the conventional hPSCs mostly in combination with H3K9me3 in the regions enriched for CpG islands (fig. S11D). Furthermore, regions marked by a combination of H3K27me3/H3K9me3 in capacitated cells but by H3K9me3 only in the conventional cells were enriched in repeats and retrotransposons (Fig. 6F and fig. S11E). The regions solely marked by H3K27me3 in the capacitated cells but by H3K9me3 in the conventional cells were enriched in repeats and Lamin B1 binding regions. It has been shown that *Xist* directly interacts with the Lamin B receptor in mice and recruits the inactive X to the nuclear lamina, enabling efficient gene silencing (*46*). The regain of H3K27me3 domains in LaminB1-binding regions in our capacitated cells might reflect the gene inactivation through *XIST*-dependent recruitment of the X chromosome to the nuclear lamina. However, the role of the Lamin B receptor in X chromosome silencing is still under debate (*47*, *48*), and our results cannot resolve this. More experimental evidence will be needed to determine the functional role of Lamin B1 in X chromosome silencing of capacitated and conventional hPSCs.

Last, we validated the levels and distribution of H3K27me3, *XIST*, and *XACT* by immuno–fluorescence in situ hybridization (immuno-FISH) (Fig. 6, G and H, and fig. S11F) and the expression of *XIST* by qualitative polymerase chain reaction (qPCR) (Fig. 6I). Conventional primed H9-EOS showed nearly completely eroded X chromosome state (91.3 to 93.0% cells), featuring biallelic *XACT* expression and lack of *XIST* and H3K27me3. A round of resetting and capacitation nearly fully restored X inactivation (88.0 to 92.5% cells), with inactive X chromosomes marked by *XIST* and H3K27me3, with a proportion of those simultaneously carrying *XACT*. However, capacitated cells progressively accumulated a subpopulation with eroded X chromosomes after extended passaging (20.0 to 27.0% in d20X and 5.9 to 14.0% in d20E cells). We analyzed *XIST* levels in the capacitated cells after further passaging for 50 days and found a complete loss in XAF conditions, suggesting complete erosion. However, the cells in E8 stably maintained *XIST* expression similar to d20E cells.

Our results, based on the epigenetic differences and *XIST*/*XACT* levels between capacitated and conventional hPSCs, suggest that a round of resetting and capacitation reinstated X chromosome inactivation, reversing culture-induced X chromosome erosion.

## DISCUSSION

**Fig. 7.**
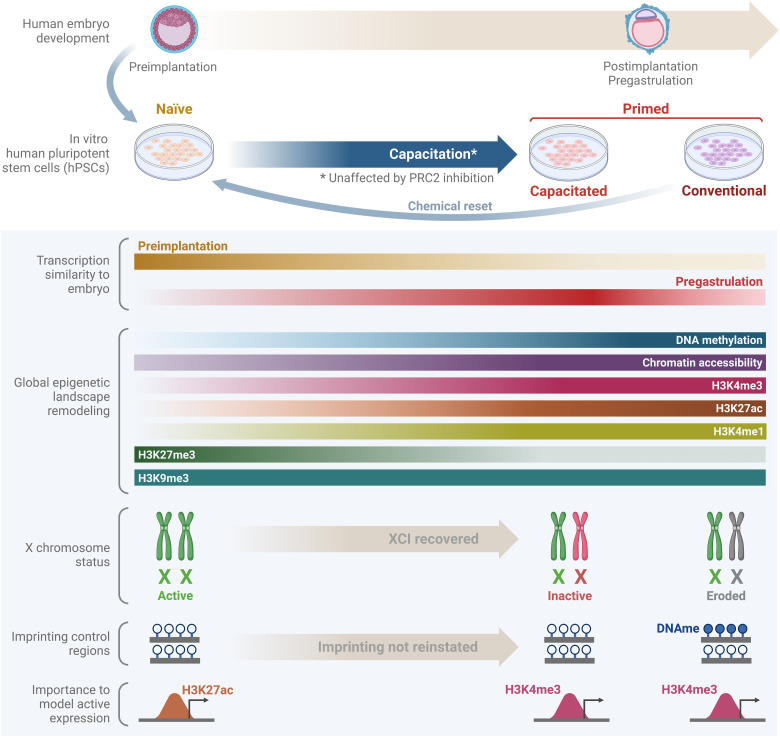
Epigenetic dynamics during capacitation. Naïve hPSC can be derived from preimplantation hICM cells or through (chemical) resetting of conventional hPSCs. Naïve hPSC can be capacitated, a process for which PRC2 activity appears to be dispensable. While naïve hPSCs remain transcriptionally close to preimplantation epiblast cells, capacitated hPSCs are transcriptionally closer to postimplantation epiblast than conventional hPSCs. During capacitation, the hPSC epigenetic landscape is globally remodeled, including genome-wide DNA methylation increase or H3K27me3 decrease. While genomic imprinting is not maintained in naïve hPSC nor re-established during capacitation, X chromosome erosion, frequently observed in conventional female hPSCs, is reversed by resetting and subsequent capacitation. The roles of H3K27ac and H3K4me3 in gene expression control appear to be distinct between the naïve and the primed hPSCs. XCI, X chromosome inactivation; DNAme, DNA methylation.

The development of the first protocol for the derivation of hPSCs (*49*) opened up new prospects for translational research and cell-based therapies. Later, these hPSCs were assigned to the primed state correspondent to the postimplantation epiblast (*18*, *28*, *34*, *50*). However, conventional primed hPSCs display variable differentiation abilities (*51*, *52*). Moreover, despite being overall genetically stable (*50*), primed hPSCs often accumulate epigenetic aberrations during long-term maintenance (*20*–*23*). The derivation of hPSCs in developmentally earlier naïve state offered an opportunity to model human pre- and peri-implantation embryogenesis that is practically inaccessible for studies due to technical and ethical limitations (*9*, *18*, *28*, *34*). Naïve hPSCs require an additional step of capacitation before the induction of somatic lineages (*9*), which may potentially hamper their utility. Nevertheless, this system raised hopes to avoid the limitations of the primed hPSCs. In this work, using transcriptomics datasets covering an extended period of epiblast progression, we mapped hPSCs to primate developmental timeline and found that while naïve hPSCs represented preimplantation epiblast, the primed hPSCs corresponded to the postimplantation pregastrulation stage. Capacitated hPSCs recapitulated the transcriptional state of the embryonic postimplantation epiblast more faithfully than the conventional hPSCs. This motivated us to investigate the differences between different populations of hPSCs at the epigenetic level. While major epigenetic marks of the naïve and conventional primed hPSCs have been previously described and compared (*10*, *17*, *18*, *29*), capacitated hPSCs have received less attention. Here, we systematically profiled epigenetic dynamics during capacitation and compared capacitated and conventional primed hPSCs (Fig. 7).

Our results showed that, at the global level, capacitated and conventional primed hPSCs have similar epigenetic characteristics. They reinforce that there is a genome-wide increase in DNA methylation levels with CpG islands remaining low or unmethylated during the naïve-to-primed transition. Simultaneously, H3K27me3 levels substantially decreased between the two states, mainly over repetitive elements, while becoming enriched in CpG islands and surrounding regions. Furthermore, regions with decreasing accessibility during the naïve-to-primed transition were enriched for putative binding motifs of transcription factors involved in the specification of extraembryonic lineages, such as TFAP2A and TFAP2B (*53*, *54*) as well as TFAP2C, associated with both extraembryonic differentiation and naïve state identity (*55*, *56*). This reflects the recently shown capacity of naïve hPSCs to differentiate to trophectodermal lineage, which is lost during capacitation (*57*–*59*). The regions gaining accessibility in the primed hPSCs were enriched for the OCT4-SOX2 heterodimer motif and associated with organ morphogenesis genes. This is consistent with the notion that the OCT4-SOX2 heterodimer, or heterodimer configurations, has a functional role in establishing the regulatory conditions for embryo development and differentiation, apart from its role in the induction and maintenance of pluripotency (*60*).

Global shifts in the levels of major epigenetic modifications including H3K4me3, H3K27me3, H3K27ac, and DNA methylation during capacitation prompted us to examine the association between epigenetic marks and gene expression and compare the potential regulatory roles of these marks in naïve and primed hPSCs. From our analysis, transcription of genes expressed higher in the naïve state showed a higher association with H3K27 acetylation levels in their promoters, while primed-specific genes were more associated with H3K4 trimethylation levels. The global rewiring of the epigenetic landscape and changes in these histone modification levels might underlie the necessity for this switch. Furthermore, these histone modification level shifts could be partially explained by differences in the metabolism and signaling pathways between pluripotency states (*61*). High CpG density promoters were a common feature of genes that remained bivalent during capacitation, including selected lineage-specific genes. This suggests a regulatory role of histone modifications in the activation of developmental genes, consistent with previous observations (*30*). Promoters of these genes were likely evolutionarily selected to have low deamination or mutation rates, with epigenetic mechanisms having a potential role in their protection (*62*, *63*).

Global reduction and redistribution of H3K27me3 to gene regulatory elements were one of the most pronounced epigenetic changes during capacitation. H3K27me3 has been suggested to represent a roadblock during conversions between the naïve and the primed pluripotency and between pluripotency and lineage commitment (*19*, *30*, *31*). We chemically inhibited EZH2 catalytic activity to probe the relevance of H3K27me3 levels independent of other potential PRC2 functions. Unexpectedly, we observed that capacitation was not affected by the inhibition of PRC2. This contrasts with trophectoderm induction from the naïve hPSCs and endo- and mesodermal early differentiation from the primed hPSCs, which were facilitated by inhibition or elimination of PRC2 (*19*, *30*, *31*). A notable difference between endo- and mesodermal differentiation and capacitation is the role of signals. While endo- and mesodermal differentiation requires exogenous instruction, including WNT and Activin/NODAL, capacitation is triggered by the withdrawal of factors for self-renewal of the naïve hPSCs and represents an autonomous cellular program (*9*, *59*). This is reminiscent of neuroectodermal induction from primed hPSCs that occurs upon signaling inhibition (*64*), which is also not enhanced by the removal of PRC2 in EZH2 knockout (*30*). Thus, we propose a refined model of the PRC2 role, whereby it establishes a roadblock for signal-responsive lineage-specific genes to prevent their premature activation and/or to enable a regulatable switch, while cell-intrinsic autonomous developmental programs are PRC2 independent to enable their progression once self-renewal factors are withdrawn. On a special note, trophectoderm differentiation in vitro occurs upon inhibition of signaling pathways and yet is facilitated by PRC2 inhibition. Nevertheless, unlike somatic lineages, trophectoderm segregates earlier than the epiblast emergence in development, and thus PRC2-imposed block in hPSCs may reflect the inactivation of regulatory elements after this lineage decision that has occurred in the past.

Long-term in vitro culturing is often associated with the accumulation of epigenetic aberrations. For example, naïve hPSCs frequently lose DNA methylation in imprinted regions (*29*), as we observed. Here, we explored another case of epigenetic regulation, X chromosome inactivation in female cells. X chromosome erosion commonly occurs in in vitro–cultured primed female hPSCs (*20*–*22*) and represents a limitation for using female hPSCs in translational and fundamental research. Through the analysis of H3K27me3, H3K9me3, *XIST* and *XACT* levels, and nuclear localization, we found that the eroded state of the X chromosome (*20*) in long-term conventional primed hPSCs cultures was nearly fully reversed by resetting and capacitation and recovered the expected characteristics of the inactive X chromosome, including accumulation of H3K27me3 and *XIST* coating. Published data indicate nonrandom inactivation of X chromosome during differentiation of the naïve hPSCs (*43*), but we were not able to assess whether X chromosome inactivation was random from our data due to nonclonal nature of our hPSC lines. Moreover, although the capacitated hPSCs progressively accumulated cells with eroded X chromosomes during prolonged culture, we showed that using E8 medium allowed them to maintain *XIST* expression at least 50 days after capacitation, which was considerably longer than in XAF. This further confirms that capacitated hPSCs represent not only transcriptional features of embryonic postimplantation epiblast more faithfully than the conventional primed cells but also some epigenetic characteristics. Resetting followed by capacitation represents a useful method to reduce hPSCs and iPSC epigenetic variability and study X chromosome inactivation.

To conclude, we generated and analyzed a comprehensive resource with global profiling of active and repressive histone modifications, DNA methylation, chromatin accessibility, and transcription in both pluripotency states. We believe that this analysis will serve as a valuable resource for future research aiming to characterize pluripotency and uncover molecular mechanisms involved in human embryogenesis. However, further experiments are needed to uncover the causal relationship between epigenetics and pluripotency. Future research focusing on enhancer-promoter interactions, perturbation of epigenetic marks, cell heterogeneity, the role of retrotransposons, and putative intermediate pluripotent states will greatly improve our understanding of epigenetic mechanisms in the early stages of human development.

## MATERIALS AND METHODS

### Cell culture

#### Cell lines

The experiments were conducted using the embryo-derived HNES1 and the chemically reset cR-H9-EOS naïve hPSC lines (*10*, *65*), as well as conventional primed H9 hPSC (WA09, WiCell). HNES cells were derived with informed consent under license from the Human Embryology and Fertilisation Authority.

#### hPSC maintenance

Naïve hPSCs were cultured on irradiated mouse embryonic fibroblasts (MEFs) in PDLGX medium prepared as following: N2B27 supplemented with 1 μM PD032590, human leukemia inhibitory factor (10 ng/ml; both from Cambridge Stem Cell Institute facility), 2 μM Gö6983 (Tocris Bio-Techne, catalog no. 2285), and 2 μM XAV939 (Tocris Bio-Techne, catalog no. 3748), as described previously (*9*, *66*). N2B27 basal medium was prepared as follows: Neurobasal (catalog no. 21103049, Thermo Fisher Scientific) and Dulbecco’s modified Eagle’s medium (DMEM)/F12 (catalog no. 31331093, Thermo Fisher Scientific) in the ratio 1:1; 0.5% N2 (catalog no. 17202048, Thermo Fisher Scientific), 1% B27 (catalog no. 17504044, Thermo Fisher Scientific), 2 mM l-glutamine (catalog no. 25030024, Thermo Fisher Scientific), and 100 μM 2-mercaptoethanol (catalog no. M7522, Sigma-Aldrich). Naïve hPSCs were routinely passaged using TrypLE Express (catalog no. 12604021, Thermo Fisher Scientific). Geltrex (0.5 μl/ml; A1413302, Thermo Fisher Scientific) was added to the culture medium during replating. ROCK inhibitor (10 μM; Y-27632, catalog no. 688000, Millipore) was added for 24 hours after passaging. (*9*, *66*).

Conventional primed H9 hPSCs were cultured in E8 medium [prepared in-house according to Chen *et al.* (*67*)]. Conventional primed H9 hPSCs were cultured in E8 medium [prepared in-house according to Chen *et al.* (*67*)] on Geltrex precoated plates and passaged using 0.5 mM EDTA in phosphate-buffered saline (PBS). All cells were cultured in a humidified incubator with 5% O_2_ and 5% CO_2_ at 37°C.

#### Capacitation

Capacitation was done as described previously (*9*). Before capacitation, naïve hPSCs were passaged once to noncoated tissue culture plates in PDLGX medium supplemented Geltrex at 1 μl/cm^2^ to reduce the number of feeder cells. For capacitation, cells were dissociated with TrypLE and plated to Geltrex-coated tissue culture plates at a seeding density of 1.6 × 10^4^/cm^2^ in PDLGX supplemented with 10 μM ROCK inhibitor. After 48 hours, cells were washed with DMEM/F12 supplemented with 0.1% bovine serum albumin (BSA) and the medium was changed to N2B27 supplemented with 2 μM XAV939 (Tocris Bio-Techne, catalog no. 3748). The medium was refreshed every 1 to 2 days. Cells were passaged at a 1:2 ratio at confluency using TrypLE and 10 μM ROCK inhibitor.

For expansion after 10 days of capacitation, cells were cultured in either E8 or N2B27 supplemented with 2 μM XAV939, Activin A (3 ng/ml) and FGF2 [10 ng/ml; XAF medium, modified from (*68*)]. During expansion, cells were cultured on Geltrex precoated tissue culture plates and passaged by dissociation with either 0.5 mM EDTA or TrypLE. ROCK inhibitor (10 μM) was added for 24 hours after passaging. (*68*).

#### PCR2 inhibition

Cell permeable EZH2 inhibitor UNC1999 (Cayman Chemical, catalog no. 14621) was used at concentrations 1, 1.75, or 2.5 μM. Short-term inhibition was done for 4 days. and long-term inhibition was done for 14 to 21 days. For capacitation, the inhibitor was applied 4 days before the beginning of the transition and then maintained throughout the protocol.

### Data generation

#### Chromatin immunoprecipitation sequencing

A total of 5 × 10^5^ to 10^6^ cells were used for ChIP per sample. Cross-linking was done with 1% formaldehyde in DMEM added directly to cells in culture dishes for 8 min at room temperature. Quenching was performed using 0.1 M glycine (final concentration). The cells were washed with ice-cold PBS and scrapped with PBS with protease inhibitor cocktail (catalog no. 11697498001, Roche). Lysis was performed in LB1 buffer [50 mM Hepes-KOH (pH 7.5), 140 mM NaCl, 1 mM EDTA, 10% glycerol, 0.5% Igepal CA-630, 0.25% Triton X-100, and protease inhibitors] by rotating for 10 min at 4°C, followed by centrifugation at 2000*g* for 5 min. The pellet was incubated in LB2 buffer [10 mM tris-HCl (pH 8.0), 200 mM NaCl, 1 mM EDTA, 0.5 mM EGTA, and protease inhibitors] by rotating for 5 min at 4°C, followed by centrifugation at 2000*g* for 5 min. The pellet was resuspended in a buffer containing 50 mM tris-HCl (pH 8.0), 10 mM EDTA, and 1% SDS, and the sonication was performed for 45 cycles and 30-s intervals on/off. Debris was removed by centrifugation, and 10% from the sonicated material was saved as input.

Before immunoprecipitation, 5 μg of antibody and 100 μl of protein A beads were preincubated overnight at 4°C in a total volume of 250 μl, adjusted with PBS with BSA (5 mg/ml), followed by three washes using the same solution. Immunoprecipitation was done by combining the sonicated material with the bead-antibody complexes, in a total volume of 400 μl adjusted with ChIP dilution buffer [10 mM tris-HCl (pH 8.0), 100 mM NaCl, 1 mM EDTA, 0.5 mM EGTA, 0.1% sodium deoxycholate, 0.5% *N*-lauroylsarcosine, 1% Triton X-100, and protease inhibitors], by rotating overnight at 4°C. After the incubation, the beads were washed six times with radioimmunoprecipitation assay buffer [50 mM Hepes-KOH (pH 7.5), 500 mM LiCl, 1 mM EDTA, 1% Igepal CA-630, and 0.7% sodium deoxycholate] followed by one wash in TE [10 mM tris-HCl (pH 8.0) and 1 mM EDTA]. Reverse cross-linking was done in elution buffer containing 1% SDS, 100 mM sodium bicarbonate, and 200 mM NaCl at 65°C for 9 to 15 hours. The input sample was also reverse cross-linked. After this step, the beads were removed from the mixture, and the remaining DNA in the solution was treated with 8 μg of ribonuclease A for 1 hour at 37°C, followed by 80 μg of proteinase K for 2 hours at 55°C. DNA was purified using MinElute columns and then used for ChIP-seq library preparation using the NEXTflex Rapid DNA-Seq Kit (NOVA-5144-02) according to the manufacturer’s protocol with some minor modifications. Briefly, we amplified the final libraries with four cycles of PCR, then performed an AMPure XP-based size selection (0.5× to eliminate larger fragments followed by 1.8× to extract the smaller fragments), and continued with eight more PCR cycles on the size-selected material. The final libraries were then cleaned up with 0.8x vol AMPure XP beads. Sequencing was carried out on HiSeq 2500 instruments (Illumina).

#### Assay for transposase accessible chromatin with sequencing

To isolate nuclei, 5 × 10^4^ cells were resuspended in 50 μl of cold lysis buffer [10 mM tris-HCl (pH 7.4), 10 mM NaCl, 3 mM MgCl_2_, and 0.1% Igepal C-630], and the mixture was pipetted up and down 16 times and immediately centrifuged at 500*g* for 10 min at 4°C. The pellet was used for tagmentation reaction, which was performed with 2.5 μl of TDE1 in 50-μl total volume (FC-121-1031, Nextera DNA Library Prep Kit, Illumina). The samples were then further amplified (Nextera kit FC-121-1030) and barcoded with the corresponding indices (FC-121-1011). The final libraries were then cleaned up with 1.2x volume of AMPure XP beads. Sequencing was carried out on a NovaSeq 6000 instruments (Illumina).

#### Whole-genome bisulfite sequencing

PBAT libraries for whole-genome DNA methylation analysis were prepared from purified genomic DNA as previously described (*69*–*71*). Paired-end sequencing was carried out on a HiSeq 2500 instruments (Illumina).

#### Karyotype analysis

Slides for metaphase G-banding were prepared using standard techniques (*72*). Briefly, 50% confluent hPSCs were incubated with KaryoMAX Colcemid (80 ng/ml) for 4 hours (Thermo Fisher Scientific, catalog no. 15212012). The cells were harvested using TrypLE, washed, and treated with hypotonic 37.5 mM KCl solution for 5 min at 37°C, followed by three rounds of fixation with Carnoy’s fixative (acetic acid:methanol in the ratio 1:3). One drop of suspension was dropped onto a glass microscope slide (Sigma-Aldrich). (*72*).

G-banding was performed by exposure to 0.12% trypsin-EDTA dissolved in Sorenson’s buffer (6.7 mM KH_2_PO_4_ and 6.7 mM Na_2_HPO_4_) for 25 s. Metaphases were stained by incubation at room temperature with Leishmann’s dye (0.18% in methanol) in Gurrs buffer (1:4; Sigma-Aldrich) for 2 min. G-banded slides were scanned, and metaphases were captured and analyzed using a CytoVision GSL-120 (Leica Microsystems) image analysis system. Metaphase analysis of between 5 and 45 cells was performed by a Health Professionals Council registered Clinical Scientist in a United Kingdom Accreditation Service (UKAS) accredited laboratory.

#### Western blot analysis

Histone protein acid extracts (Abcam Protocol) were resolved using a gradient SDS–polyacrylamide gel electrophoresis before being immunoblotted onto a polyvinylidene difluoride or nitrocellulose membrane. The membrane was blocked for 1 hour in blocking solution [tris-buffered saline (TBS)/0.1% Tween/5% BSA] and then incubated overnight at 4°C with primary antibodies diluted in blocking solution. After washes with TBS–0.1% Tween, the membranes were incubated with secondary antibodies diluted in blocking buffer for 1 hour at room temperature. The horseradish peroxidase (HRP) conjugates were detected using an enhanced chemiluminesence solution, which generates chemiluminescence. The following primary antibodies were used: for Fig. 2 and fig. S4: H3 (1:1000; Abcam, #ab1791), H3K4me1 (1:1000; Abcam, #ab8895), H3K4me3 (1:1000; Abcam, #ab8580), H3K27me3 (1:1000; Cell Signaling Technology, #C36B11), H3K27ac (1:1000; Abcam, #ab4729), and H3K9me3 (1:1000; Abcam, #ab8898) and for Fig. 3: H4 (1:1000; Cell Signaling Technology, #13919) and H3K27me3 (1:1000; Cell Signaling Technology, #9733). The secondary antibody used were an HRP-conjugated monoclonal donkey anti-rabbit immunoglobulin G (1:5000; Amersham, #NA934).

#### Immuno-FISH

The cells grown on glass cover slips were fixed with 3% paraformaldehyde in PBS for 10 min, washed twice with PBS, and permeabilized for 5 min in PBS containing 0.1% Triton X-100, followed by additional three washes, all at room temperature. The coverslips were stored at 4°C for 5 to 7 days.

The coverslips were incubated with the H3K27me3 antibody (0.4 μg/ml; Active Motif, #39155) in PBS for 1 hour at room temperature, then washed three times for 10 min with PBS, followed by a 1-hour incubation with an Alexa-488–labeled goat anti-rabbit antibody (0.8 μg/ml; Alexa-488 A11008 Thermo Fisher Scientific) in the dark. After three washes with PBS, the cells were fixed again with 3% paraformaldehyde in PBS for 10 min at room temperature, followed by three short washes with PBS and two washes with 2× SSC.

RNA-FISH was performed using a bacterial artificial chromosome (BAC) probe against human *XACT* (BAC RP11-35D3) and a plasmid (vi.34) probe containing the human *XIST* cDNA sequence (*73*) as described previously with minor modifications (*74*). The *XACT* and *XIST* probes were labeled by nick translation (Abbot) using deoxyuridine triphosphate (dUTP)-Atto550 (Jena Bioscience) and Cy5-dUTP (Cytiva).

Per coverslip, 200 ng of *XACT* probe was ethanol-precipitated with salmon sperm DNA (Roche, 11467140001) and Cot1 repeats (Thermo Fisher Scientific, catalog no. 18440016), resuspended in formamide for 30 min at 37°C, denatured (10 min, 75°C), and competed for 1 hour at 37°C. The *XIST* plasmid probe was precipitated without Cot1, and the competition step was omitted. Both probes were cohybridized in hybridization buffer [50% formamide, 20% dextran sulfate (Sigma-Aldrich, catalog no. D8906), BSA (2 mg/ml; NEB, catalog no. B9000S), 2× SSC (Sigma-Aldrich, catalog no. S6639)] at 37°C overnight in the dark. Coverslips were washed three times for 7 min at 42°C with 2× SSC/50% formamide (pH 7.2), followed by three washes with 2× SSC for 5 min at 42°C. DNA staining was done by incubating the cells in DAPI (0.3 μg/ml) in 2× SSC buffer for 3 min at room temperature, followed by three washes with 2× SSC. The coverslips were then mounted with VECTASHIELD (Vectorlabs, catalog no. H-1000) and sealed with nail polish. Images were acquired using a widefield Z1 Observer microscope (Zeiss) using a 100× objective.

#### Quantitative real-time PCR

Total RNA was extracted using an RNeasy Mini kit (QIAGEN, catalog no. 74106), treated with deoxyribonuclease (Thermo Fisher Scientific, catalog no. EN0521), and reverse-transcribed with the RevertAid First Strand cDNA Synthesis Kit (Thermo Fisher Scientific, catalog no. K1622). Quantitative PCR was done using Brilliant III Ultra-Fast SYBR Green Master Mix (Agilent, catalog no. 600882) with Bio-Rad CFX384 Real-Time PCR Detection System.

#### Imaging

Brightfield images were acquired using Nikon microscope and Hamamatsu ORCA-spark digital complementary metal-oxide semiconductor (CMOS) camera. Brightfield images were acquired using Nikon microscope and Hamamatsu ORCA-spark digital CMOS camera. Acquisition and processing of images were done using HCImage (Hamamatsu) and Fiji ImageJ2 software (*75*).

### Data processing

The list of software and databases used during data processing are available in the table S15.

#### Genome build and annotation

Sequencing data raw reads from all assays were mapped to the GRCh38 primary assembly downloaded from Ensembl (release 98; download link: https://ftp.ensembl.org/pub/release-98/fasta/homo_sapiens/dna/Homo_sapiens.GRCh38.dna_sm.primary_assembly.fa.gz; link copied on the 7 December 2022). The chromosome lengths used during the analysis were obtained from the GRCh38 primary assembly FASTA file.

Genomic features were annotated using the *Homo sapiens* Ensembl gene annotation release 98 (download link: https://ftp.ensembl.org/pub/release-98/gtf/homo_sapiens/Homo_sapiens.GRCh38.98.gtf.gz; link copied on 7 December 2022) and the annotatr package from Bioconductor. Repetitive and centromeric regions were downloaded from the UCSC (University of California Santa Cruz) table browser (clade: “Mammal,” genome: “Human,” assembly: “Dec. 2013 (GRCh38/hg38)”). The repetitive regions were obtained from the track “RepeatMasker,” and table “rmsk” and centromeric regions are from the track “Centromeres” and table “centromeres.” Human naïve and primed enhancers and super-enhancers regions were downloaded from (*35*) and converted from hg19 to hg38 using a UCSC Liftover chain (download link: https://hgdownload.cse.ucsc.edu/goldenpath/hg19/liftOver/hg19ToHg38.over.chain.gz; link copied on 7 December 2022). Human imprinted regions were previously defined in the Wolf Reik research group with assistance from the bioinformatics facility at the Babraham Institute. The blacklisted regions used in the ChIP-seq and ATAC-seq alignment pipelines were the hg38 ENCODE blacklist version 2 downloaded from the Boyle Lab (*76*).

#### Read alignment and quantification

The reads from each assay were aligned and quantified employing the community-curated nf-core pipelines. The single-end RNA-seq data were processed using the “rnaseq” pipeline (version 1.4.2) with the pseudo-aligner Salmon selected; the ChIP-seq data (histone modifications H3K4me1, H3K4me3, H3K9me3, H3K27me3, and H3K27ac) were processed using the “chipseq” pipeline (version 1.1.0) with the Burrows-Wheeler Aligner (BWA) and MACS2 peak caller being chosen as default. Histone modifications H3K4me3 and H3K27ac peaks were called in the MACS2 “narrowPeak” mode, while the histone modifications H3K4me1, H3K27me3, and H3K9me3 peaks were called in the MACS2 “broadPeak” mode. The input control used for peak calling in all samples was the merged purified DNA sequencing data from cR-H9-EOS cells at day 0, cR-H9-EOS cells at day 10, and the conventionally cultured (E8 medium) H9 hPSCs (each condition with two biological replicates). The ATAC-seq data were processed using the “atacseq” pipeline (version 1.1.0) with the BWA and MAC2 peak caller selected as default. The ATAC-seq peaks were called in the MACS2 narrowPeak mode. The PBAT data were processed using the “methylseq” pipeline (version 1.4), with Bismark selected for mapping and methylation calling.

#### RNA-seq gene read counts

The RNA-seq read counts obtained from the Salmon transcript quantification were imported into R and converted to gene read counts using the tximport package. The gene read counts were subsequentially transformed using the variance stabilizing transformation from DESeq2 for the correlation and clustering plots. For the remaining plots, the reads were normalized by estimating the size factors and retrieving the normalized counts with DESeq2. For the differentially expressed gene analysis, raw gene read counts were used.

#### ChIP-seq and ATAC-seq read counts in genomic regions

The ChIP-seq and ATAC-seq aligned read data were imported into R, and the number of reads that overlap genomic regions (i.e., genomic windows and features) was counted with the function “summarizeOverlaps” from the Bioconductor package GenomicAlignments with the parameter inter.feature set as “FALSE,” the parameter ignore.strand set as “TRUE,” and the remaining parameters kept as default. The read counts were transformed using the variance stabilizing transformation from DESeq2 and used as input for the correlation plots. For the remaining plots, the DESeq2 normalized counts were used instead.

#### Methylation percentage in CpGs and genomic regions

While running the nf-core methylseq pipeline, Bismark generated a coverage file with the PBAT sequencing read information for each cytosine. Those files were imported into R, and with the methylKit package, only read count data in a CpG context were selected. Also, to exclude cytosines with an extremely high number of read counts, positions belonging to the top 0.1 percentile of read counts were excluded. The normalized coverage values between samples were calculated by a scaling factor derived from differences between the median of coverage distributions using the “normalizeCoverage” function from methylKit. Because several samples had low genome-wide coverage, the reads from all replicates in each CpG position were pooled, and only the CpGs with three or more pooled reads were selected. The methylation percentage in each CpGs was calculated by dividing the number of Cs (cytosines) by the CpG coverage, and the result was multiplied by 100. The read counts over genomic regions were determined using the function “regionCounts” function from methylKit. The function filtered out regions with less than two bases covered or regions with an overall coverage of fewer than three reads. The methylation percentage of those genomic regions was also calculated by pooling the number of Cs and dividing that number by the CpG coverage, and the result was multiplied by 100.

#### Differentially expressed genes and clusters

The RNA-seq raw read counts were used to compute the differentially expressed genes using the package DESeq2 with the design formula having day in culture as the only variable. The differential genes were obtained for each day in culture and cell type. After computing the differentially expressed genes for each condition, only genes with an adjusted *P* value below 0.001 and a log_2_ fold change of more than 2.5 were selected. The differentially expressed genes between every condition combination were clustered using the package Mfuzz with eight cluster centers and 0.9 as the minimum membership.

#### Differential ChIP-seq and ATAC-seq peaks

The peaks differentially enriched between conditions were determined using the R Bioconductor package DiffBind. Peaks from ATAC-seq, H3K27ac, and H3K4me3 ChIP-seq data were recentered around the consensus summit and resized to have 200 base pairs upstream and downstream of the center. Peaks from H3K27me3, H3K4me1, and H3K9me3 ChIP-seq data were recentered around a consensus summit and resized to have 500 base pairs upstream and downstream of the center. During the DiffBind analysis, no blacklist or graylist was applied to the DBA object.

### Data analysis

The list of software and databases used during data analysis are available in the table S15.

#### Comparison of hPSCs during capacitation to embryonic epiblast

The following embryo-derived RNA-seq datasets were used for the analysis: from in vitro–cultured human embryos [GSE136447; Xiang *et al.* (*26*)], in vitro–cultured cynomolgus macaque gastrulating embryos [GSE130114; Ma *et al.* (*25*)], and in utero human gastrulating embryo [Array Express: E-MTAB-9388; Tyser *et al.* (*27*)]. The datasets were processed using Seurat package V4.0.1 [Hao *et al.* (*77*)], as described in (*24*). The quality control–filtered and annotated single-cell RNA-seq datasets were combined using “merge” function in Seurat package, considering only epiblast cells and only protein-coding genes. One-to-one orthology was used to combine human and cynomolgus monkey data.

Bioinformatic analysis was done using RStudio software. ggplot2 package was used for data visualization. To compare the in vitro–cultured cells to the embryo, Pearson correlation coefficients were calculated between bulk RNA-seq obtained from hPSCs and pseudobulk gene expression of the embryonic epiblast subpopulations. Differential gene expression analysis was done using DESeq2 package with the significance cutoff false discovery rate (FDR) < 0.05 and abs(log_2_FC < 1). GO analysis was performed using the Enrichr web tool.

#### PCA and hierarchical clustering

The PCA and hierarchical clustering plots were generated using the differentially expressed genes between all conditions combinations from the RNA-seq data, the differential peaks between all conditions combinations from the ATAC-seq and ChIP-seq analysis with an FDR of less than 0.05 and an absolute fold change of more than 2, and the top 5% most variable 200 CpG-containing genome-wide windows for the methylation data. The 200 CpG-containing genome-wide windows were generated with the software SeqMonk.

#### Multi-omics factor analysis

MOFA2 was used to extract factors that are responsible for sample clustering using all data assays combined. The MOFA2 model was constructed using the same input as the PCA and hierarchical clustering: the differentially expressed genes from the RNA-seq data, the differential peaks from the ATAC-seq and ChIP-seq analysis with an FDR of less than 0.05 and an absolute fold change of more than 2, and the top 5% most variable 200 CpG-containing genome-wide windows for the methylation data. The model was restricted to three factors and the training to 2000 maximal interactions in the “slow” convergence mode. The t-distributed stochastic neighbor embedding plot was generated using the model output with a perplexity equal to 4.

#### Chromatin state discovery

The ChromHMM model was created using a concatenated design. First, we generated binary files using the function “BinarizeBam” from the bam files obtained after read alignment. Afterward, we used the function “LearnModel” to learn the model and the function “CompareModels” to compare models with a different number of states with a model with 40 states. The length of the segments was kept as default, i.e., 200 base pairs. Those state segments were then imported to R and used for overlap enrichment with genomic features.

#### Enriched genomic regions

The R Bioconductor package Locus Overlap Analysis (LOLA) was used to compare the enrichment of selected genomic regions with public databases of annotated regions. LOLA core and extended hg38 databases were used in the analysis. In addition, a custom database was created using the genomic annotated regions from the *H. sapiens* Ensembl gene annotation release 98 and the R Bioconductor annotatr package. It was also included to that custom database the following features: repeat classes and families, CpG island types, and promoter GC skew types. Only significant results, with an FDR adjusted *P* value of less than 0.05, were selected.

#### Multivariate regression analysis

For the regression analysis, the observed over/expected ratio (O/E ratio) of the number of CpGs in all protein-coding gene promoters (defined as TSS, ±2 kb) was first calculated using a custom script applying the function “oligonucleotideFrequency” from the Bioconductor package BSgenome. The promoters were then separated into three categories: promoters with high CpG density (O/E ratio > 0.7), promoters with medium CpG density (O/E ratio ≥ 0.35 and O/E ratio ≤ 0.7), and promoters with low CpG density (O/E ratio < 0.35). Then, the R function “boot” was used with the linear formula:

″RNA-seq″∼″ATAC-Seq″+H3K27ac+H3K27me3+H3K4me1+H3K4me3+H3K9me3+methylation

and with 1000 bootstrap replicates. For the RNA-seq data, the gene expression logarithm (log)–normalized counts were used, and for the ATAC-seq and ChIP-seq data, it used the log-normalized counts that overlapped promoters (with at least 1–base pair overlap). For the methylation data, the methylation ratio inside the promoter region was calculated as previously described for genomic windows (see the “Methylation percentage in CpGs and genomic regions” section). All datasets were rescaled to have values between 0 and 1 before the regression analysis. The relative importance of regressors was calculated using the function “boot.relimp” from the R package relaimpo. For this analysis, only protein-coding genes were used. The same methodology was applied for the regression analysis of genes belonging to the RNA-seq Mfuzz-derived clusters.

#### Gene function profiling

The GO analysis was generated using the function “enrichGO” from the Bioconductor package clusterProfiler. In all GO analyses, the database org.Hs.eg.db was used; the subontologies “biological process,” “molecular function,” and “cellular component” were selected; the adjusted *P* value procedure used was the Benjamini-Hochberg; and the *q* value had a cutoff of 0.1.

#### Bivalency and development-associated genes

Bivalent promoters were defined by selecting only the promoters (TSS ± 2 kb) containing both H3K27me3 and H3K4me3 MACS2 peaks (from the ChIP-seq dataset) using the function “subsetByOverlaps” from the Bioconductor package IRanges with parameters kept as default (with at least 1–base pair overlap). The list of developmental genes analyzed and categorized as “naïve pluripotency,” “general pluripotency,” “postimplantation epiblast,” and “lineage marker”–associated genes were obtained from (*9*).

#### Imprinting control regions allelic analysis

The human ICRs used in this analysis were previously defined in the Wolf Reik research group with assistance from the bioinformatics facility at the Babraham Institute. To perform the allelic differential analysis, the single-nucleotide polymorphisms (SNPs) from the H9 cell line (provided by the Babraham Institute bioinformatics facility) were used to assign the sequencing reads to each allele using the software SNPsplit. The reads assigned to each allele from the PBAT (with DNA methylation data), ChIP-seq (with H3K4me3, H3K4me1, H3K27ac, H3K27me3, and H3K9me3 data), and ATAC-seq assays were then imported into the software SeqMonk. The reads overlapping the ICRs were counted in each allele, and the Bioconductor package EdgeR was used to perform a statistical significance test between allele counts in each ICR. A *P* value cutoff of 0.05 was used. The results were then converted from hg19 to hg38 using a UCSC Liftover chain (download link: https://hgdownload.cse.ucsc.edu/goldenpath/hg19/liftOver/hg19ToHg38.over.chain.gz, link copied on 7 December 2022).

#### Motif enrichment

Motif enrichment analysis was performed using the software MEME-ChIP from the MEME Suite in the differential enrichment mode. The sequence alphabet was defined as “DNA, RNA, or Protein”, the primary sequences were the significant MACS2 peaks, the control sequences were the nonsignificant MACS2 peaks, and the database used was the JASPAR 2022 CORE vertebrates nonredundant. The remaining options were kept as default.

#### Transposable elements differential expression

The differential expression of transposable element subfamilies was performed using the package SalmonTE using “hs” (*H. sapiens*) as reference, and for the differential analysis, the analysis type selected was “DE.” The remaining parameters were kept as default. The RNA-seq fastq output files were used as input.
