# Supplementary material for: Epigenetic dynamics during capacitation of naïve human pluripotent stem cells
Source: Sci Adv. 2023 Sep 29;9(39):eadg1936. doi: 10.1126/sciadv.adg1936 (PMC10541016; doi:10.1126/sciadv.adg1936)
Supplement: Supplementary file 1 — Figs. S1 to S11 Legends for tables S1 to S18 [file sciadv.adg1936_sm.pdf]

Science Advances
AAAS

Supplementary Materials for

Epigenetic dynamics during capacitation of naïve human pluripotent stem cells

João Agostinho de Sousa *et al.*

Corresponding author: Ferdinand von Meyenn, ferdinand.vonmeyenn@hest.ethz.ch;
Maria Rostovskaya, maria.rostovskaya@babraham.ac.uk

Sci. Adv. **9**, eadg1936 (2023)
DOI: 10.1126/sciadv.adg1936

The PDF file includes:

Figs. S1 to S11
Legend for tables S1 to S18

Other Supplementary Material for this manuscript includes the following:

Tables S1 to S18

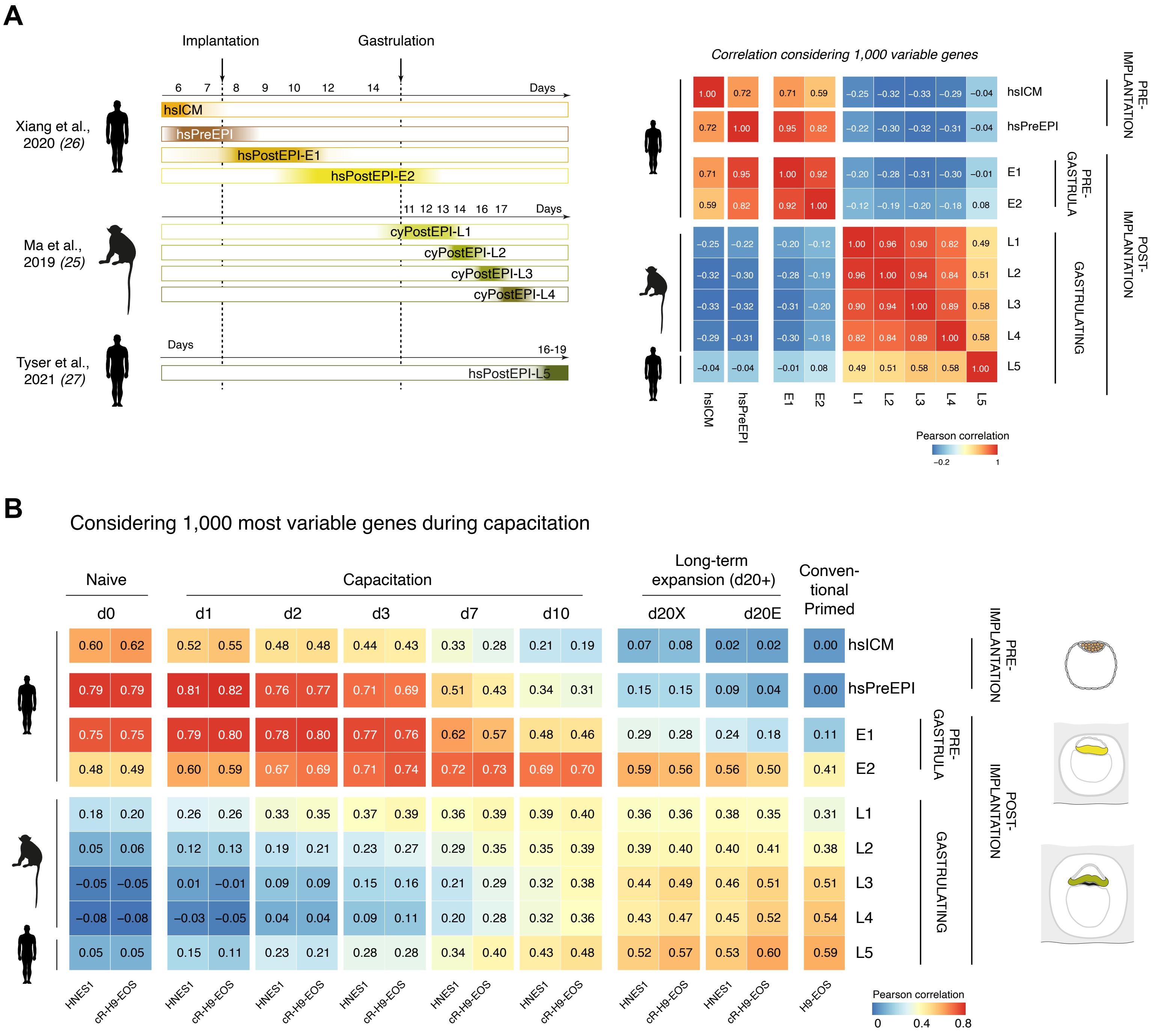

Fig. S1. Transcriptional comparison between hPSCs during *in vitro* capacitation and embryonic epiblast progression.
(**A**) Cross-correlation of stages during embryonic epiblast progression. Pearson correlation coefficients calculated using single-cell RNA-Seq data (*25–27*). (**B**) Pearson correlations between hPSCs during capacitation *in vitro* and embryonic epiblast at different developmental stages, calculated considering genes differentially expressed during the transition (included genes differentially expressed between any two samples in the dataset with FDR < 0.05 and log2 fold change > 1).

A

Original cell line	Days of capacitation	Experiment ID	Karyotype
cR-H9-EOS	20	224	46,XX[26]<br>46,XX,del(18)(q12.3q12.3)[4]
cR-H9-EOS	50	229	46,XX[29]<br>45,XX,add(4)(p1),-15,add(17)(p1)[1]

B

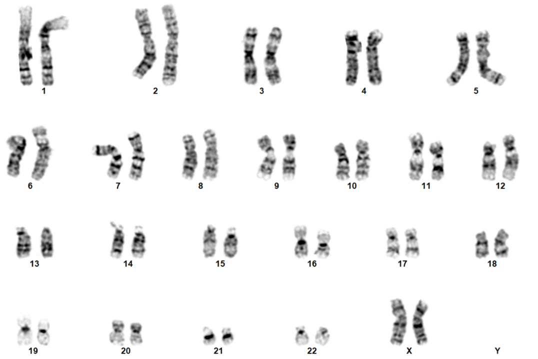

C

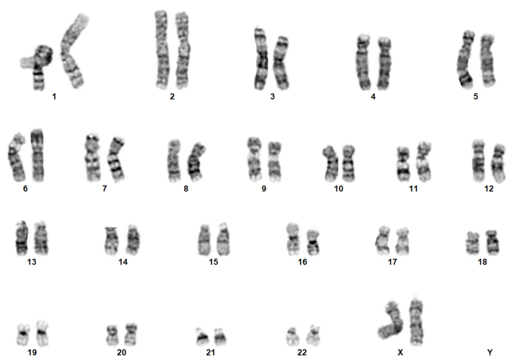

Fig. S2. Karyotype analysis of capacitated cells.
(**A**) Summary of the results. (**B**) and (**C**) Images of metaphase spreads.

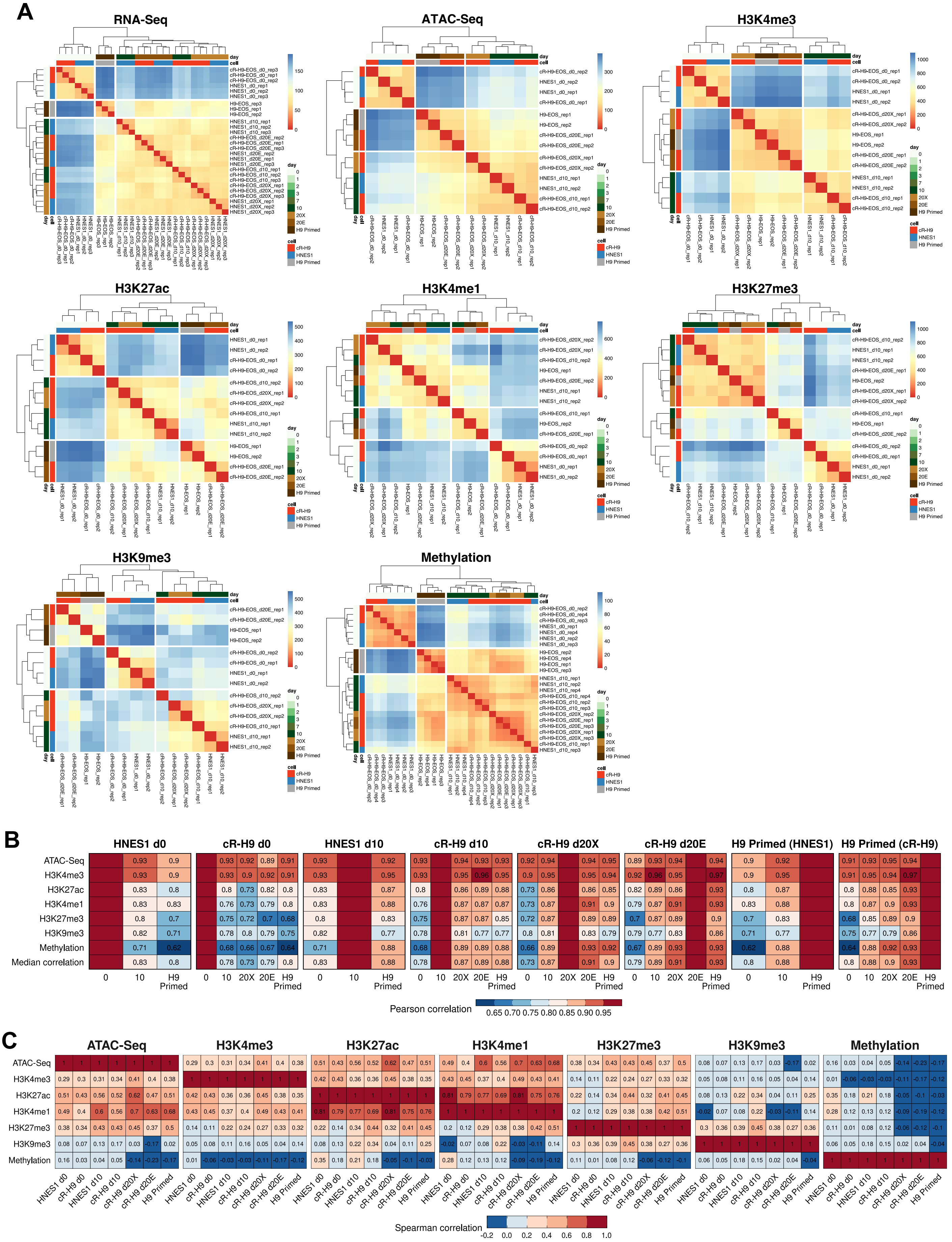
A
RNA-Seq
ATAC-Seq
H3K4me3
H3K27ac
H3K4me1
H3K27me3
H3K9me3
Methylation
day
cell
cR-H9
HNES1
H9 Primed
B
HNES1 d0
cR-H9 d0
HNES1 d10
cR-H9 d10
cR-H9 d20X
cR-H9 d20E
H9 Primed (HNES1)
H9 Primed (cR-H9)
ATAC-Seq
H3K4me3
H3K27ac
H3K4me1
H3K27me3
H3K9me3
Methylation
Median correlation
Pearson correlation
0.65 0.70 0.75 0.80 0.85 0.90 0.95
C
ATAC-Seq
H3K4me3
H3K27ac
H3K4me1
H3K27me3
H3K9me3
Methylation
HNES1 d0
cR-H9 d0
HNES1 d10
cR-H9 d10
cR-H9 d20X
cR-H9 d20E
H9 Primed
Spearman correlation
-0.2 0.0 0.2 0.4 0.6 0.8 1.0

Fig. S3. Global comparison of epigenetic characteristics between hPSCs in different conditions.

(**A**) Hierarchical clustering based on Euclidean distance between sample replicates for each sequencing assay. The clustering was generated using the top 500 most variable differentially expressed genes, histone modifications ChIP-Seq differential peaks, ATAC-Seq differential peaks, and DNA methylation average in 200 CpG-containing genomic windows between all conditions. (**B**) Pearson correlation analysis performed between one condition and the remaining conditions for each sequencing assay in 2-kb genome-wide windows. (**C**) Spearman correlation analysis performed between sequencing assays for each condition in 2-kb genome-wide windows. All results in this figure were limited to autosomes.

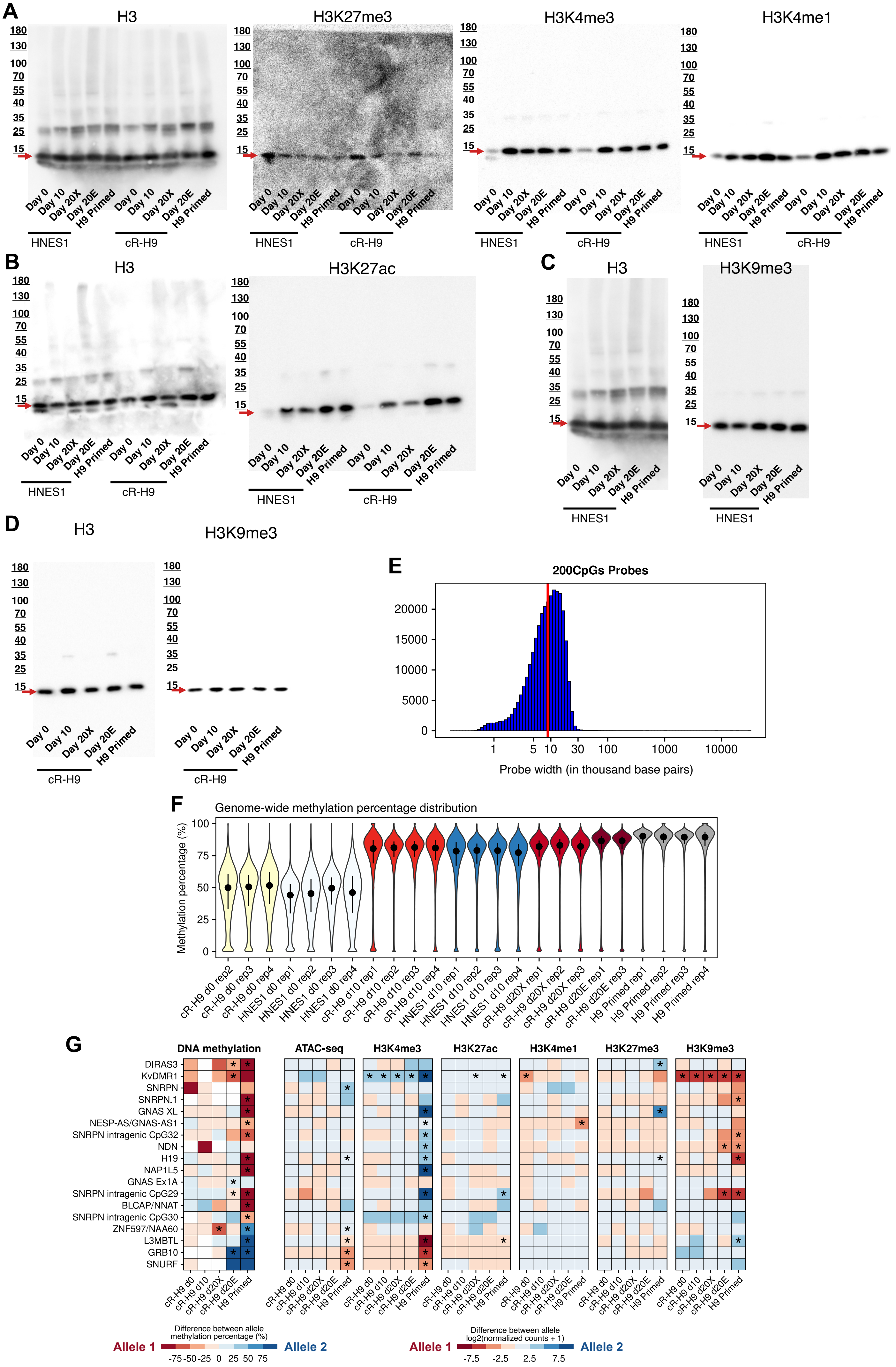

A
H3
H3K27me3
H3K4me3
H3K4me1
180
130
100
70
55
40
35
25
15
Day 0
Day 10
Day 20X
Day 20E
H9 Primed
HNES1
cR-H9
B
H3
H3K27ac
C
H3
H3K9me3
D
H3
H3K9me3
E
200CpGs Probes
20000
15000
10000
5000
0
1
5
10
30
100
1000
10000
Probe width (in thousand base pairs)
F
Genome-wide methylation percentage distribution
Methylation percentage (%)
100
75
50
25
0
cR-H9 d0 rep2
cR-H9 d0 rep3
cR-H9 d0 rep4
HNES1 d0 rep1
HNES1 d0 rep2
HNES1 d0 rep3
HNES1 d0 rep4
cR-H9 d10 rep1
cR-H9 d10 rep2
cR-H9 d10 rep3
cR-H9 d10 rep4
HNES1 d10 rep1
HNES1 d10 rep2
HNES1 d10 rep3
HNES1 d10 rep4
cR-H9 d20X rep1
cR-H9 d20X rep2
cR-H9 d20X rep3
cR-H9 d20E rep1
cR-H9 d20E rep3
H9 Primed rep1
H9 Primed rep2
H9 Primed rep3
H9 Primed rep4
G
DNA methylation
ATAC-seq
H3K4me3
H3K27ac
H3K4me1
H3K27me3
H3K9me3
DIRAS3
KvDMR1
SNRPN
SNRPN.1
GNAS XL
NESP-AS/GNAS-AS1
SNRPN intragenic CpG32
NDN
H19
NAP1L5
GNAS Ex1A
SNRPN intragenic CpG29
BLCAP/NNAT
SNRPN intragenic CpG30
ZNF597/NAA60
L3MBTL
GRB10
SNURF
cR-H9 d0
cR-H9 d10
cR-H9 d20X
cR-H9 d20E
H9 Primed
Difference between allele methylation percentage (%)
Allele 1
Allele 2
-75 -50 -25 0 25 50 75
Difference between allele log2(normalized counts + 1)
-7.5 -2.5 2.5 7.5

Fig. S4. Global epigenetic modifications levels in hPSCs.
Western blot results with antibodies recognizing: (**A**) H3, H3K27me3, H3K4me3, and H3K4me1; (**B**) H3 and H3K27ac; (**C**) H3 and H3K9me3 in HNES1 and conventional H9 cells; (**D**) H3 and H3K9me3 in cR-H9 and conventional H9 cells. (**E**) Probe width distribution of the 200 CpG probes generated with SeqMonk using the PBAT reads from all samples. (**F**) Genome-wide methylation percentage distribution in the 200 CpG probes from SeqMonk. The results from this figure were limited to autosomes. (**G**) DNA methylation and epigenetic modification allele-specific level differences in imprinting control regions (ICRs). Only the ICRs with significant results are shown in the figure. A complete list of ICRs can be found in the supplemental tables. The red-coloured boxes indicate that allele 1 had higher levels than allele 2, whereas the blue-coloured boxes indicate the opposite. The significance threshold was set to * FDR (false discovery rate) < 0.05, which was calculated using edgeR.

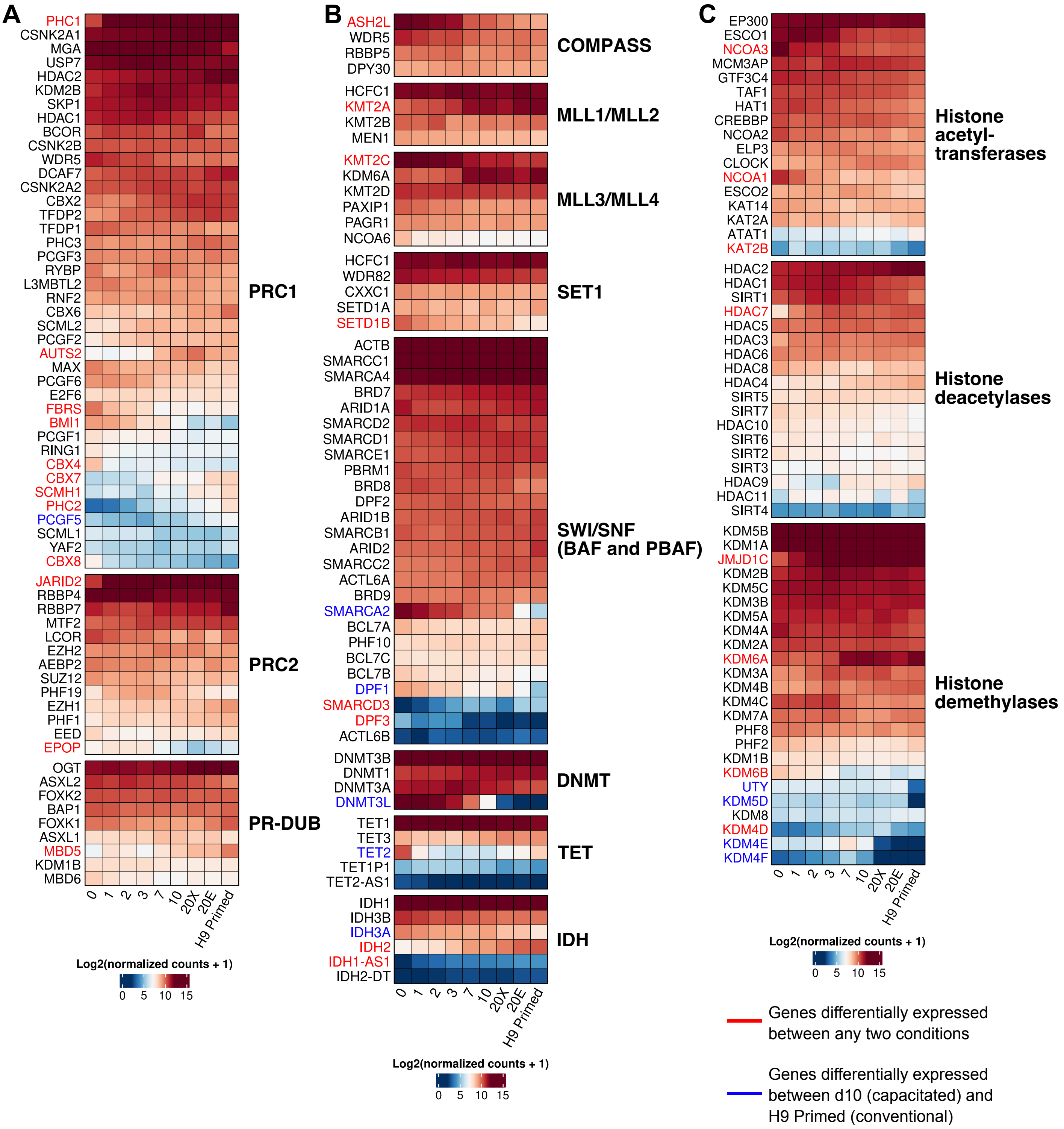

Fig. S5. Expression dynamics of chromatin modifiers.
Heatmaps with the log-normalized expression counts of genes to which their protein products belong to the Polycomb-group proteins (PcG): PRC1, PRC1, and PR-DUB complex; Trithorax-group proteins (TrxG): COMPASS, MLL1/MLL2, MLL3/MLL4, SET1, and SWI/SNF complex; the DNMT, TET, and IDH protein families; and histone acetyltransferases, histone deacetylases, and histone demethylates. The genes in red were selected for being differentially expressed between any two conditions and the genes in blue were selected for being differentially expressed between capacitated day 10 and conventional H9 primed cells.

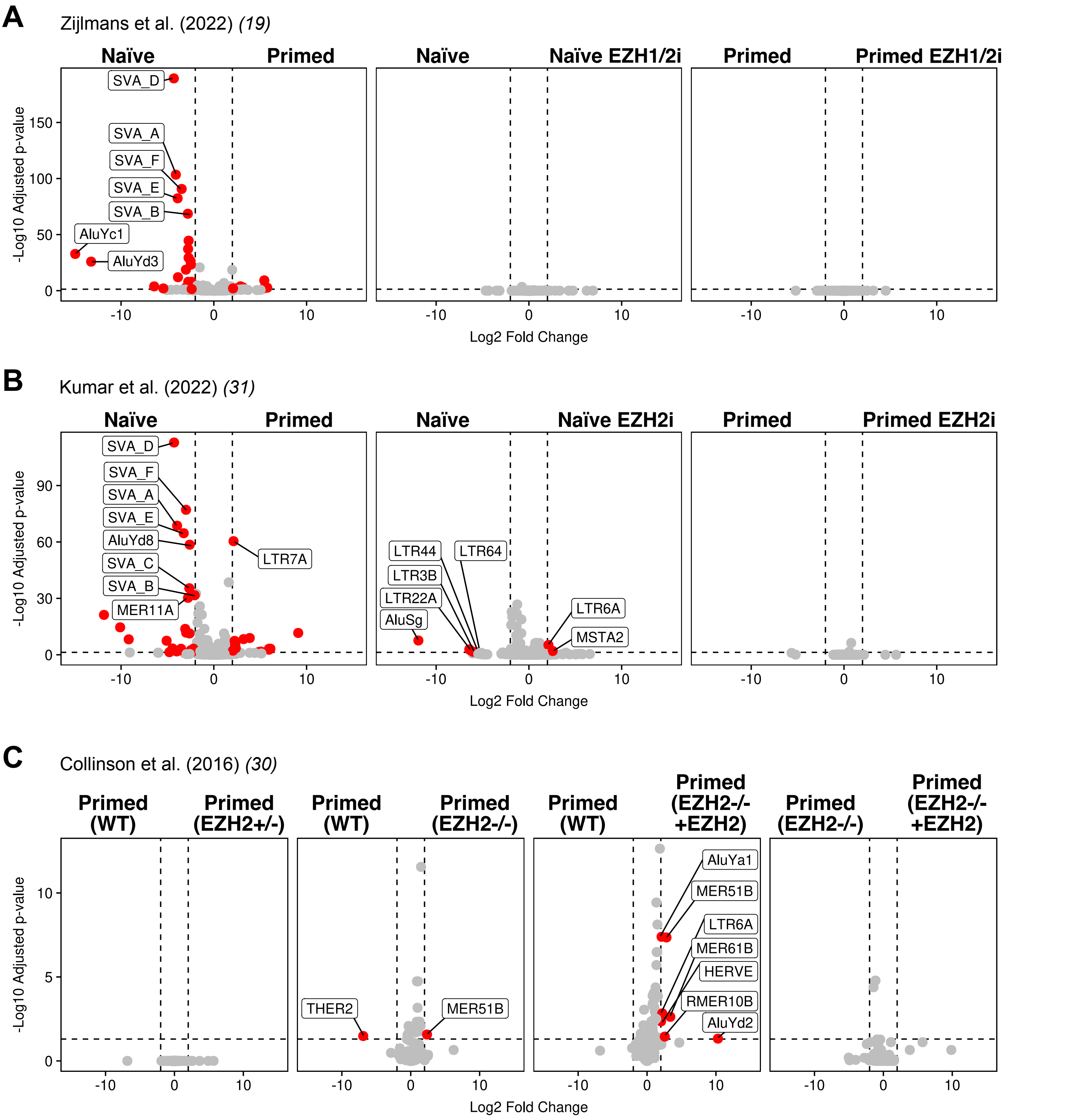

Fig. S6. Expression of transposable elements is not affected upon EZH1/2 inhibition or knockouts.
(**A**) Differential expression of transposable element classes between naïve and primed H9 cells, naïve and naïve EZH1/2i (UNC1999 inhibitor), and primed and primed EZH1/2i (UNC1999 inhibitor). The data was obtained from Zijlmans et al. (2022) (*19*). (**B**) Differential expression of transposable element classes between naïve and primed H9 cells, naïve and naïve EZH2i (media supplemented with EZSolution EPZ-6438), and primed and primed EZH2i (media supplemented with EZSolution EPZ-6438). The data was obtained from Kumar et al. (2022) (*31*). (**C**) Differential expression of transposable element classes between primed wild-type and primed EZH2+/- H9 cells, primed wild-type and primed EZH2-/-, primed wild-type and primed EZH2-/- with ectopic EZH2 expression, and primed EZH2-/- and primed EZH2-/- with ectopic EZH2 expression. The data was obtained from Collinson et al. (2016) (*30*).

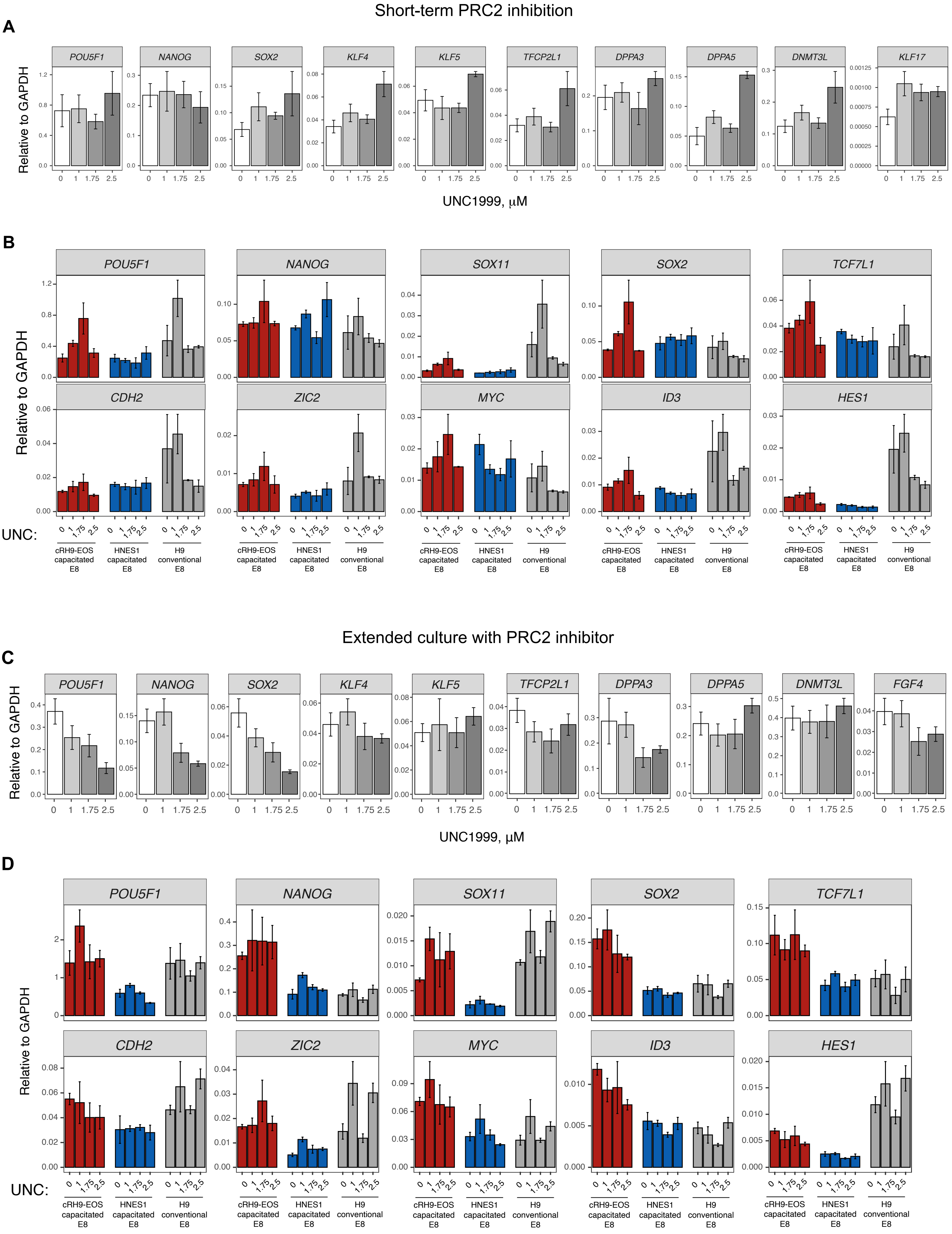
Short-term PRC2 inhibition
A
Relative to GAPDH
POU5F1
NANOG
SOX2
KLF4
KLF5
TFCP2L1
DPPA3
DPPA5
DNMT3L
KLF17
0
1
1.75
2.5
UNC1999, μM
B
Relative to GAPDH
POU5F1
NANOG
SOX11
SOX2
TCF7L1
CDH2
ZIC2
MYC
ID3
HES1
UNC:
cRH9-EOS capacitated E8
HNES1 capacitated E8
H9 conventional E8
Extended culture with PRC2 inhibitor
C
Relative to GAPDH
POU5F1
NANOG
SOX2
KLF4
KLF5
TFCP2L1
DPPA3
DPPA5
DNMT3L
FGF4
UNC1999, μM
D
Relative to GAPDH
POU5F1
NANOG
SOX11
SOX2
TCF7L1
CDH2
ZIC2
MYC
ID3
HES1
UNC:
cRH9-EOS capacitated E8
HNES1 capacitated E8
H9 conventional E8

Fig. S7. Acute PRC2 inhibition does not interfere with maintenance of hPSCs *in vitro*.
(**A**) Marker expression in the naïve hPSCs after extended culturing with PRC2 inhibitor UNC1999, shown by qRT-PCR. (**B**) Marker expression in the primed hPSCs (capacitated and conventional) after extended culturing with PRC2 inhibitor UNC1999, shown by qRT-PCR. (**C**) Marker expression in the naïve hPSCs following short-term inhibition of PRC2 activity using UNC1999 inhibitor, shown by qRT-PCR. (**D**) Marker expression in the primed hPSCs (capacitated and conventional) following acute inhibition of PRC2 activity using UNC1999 inhibitor, shown by qRT-PCR.

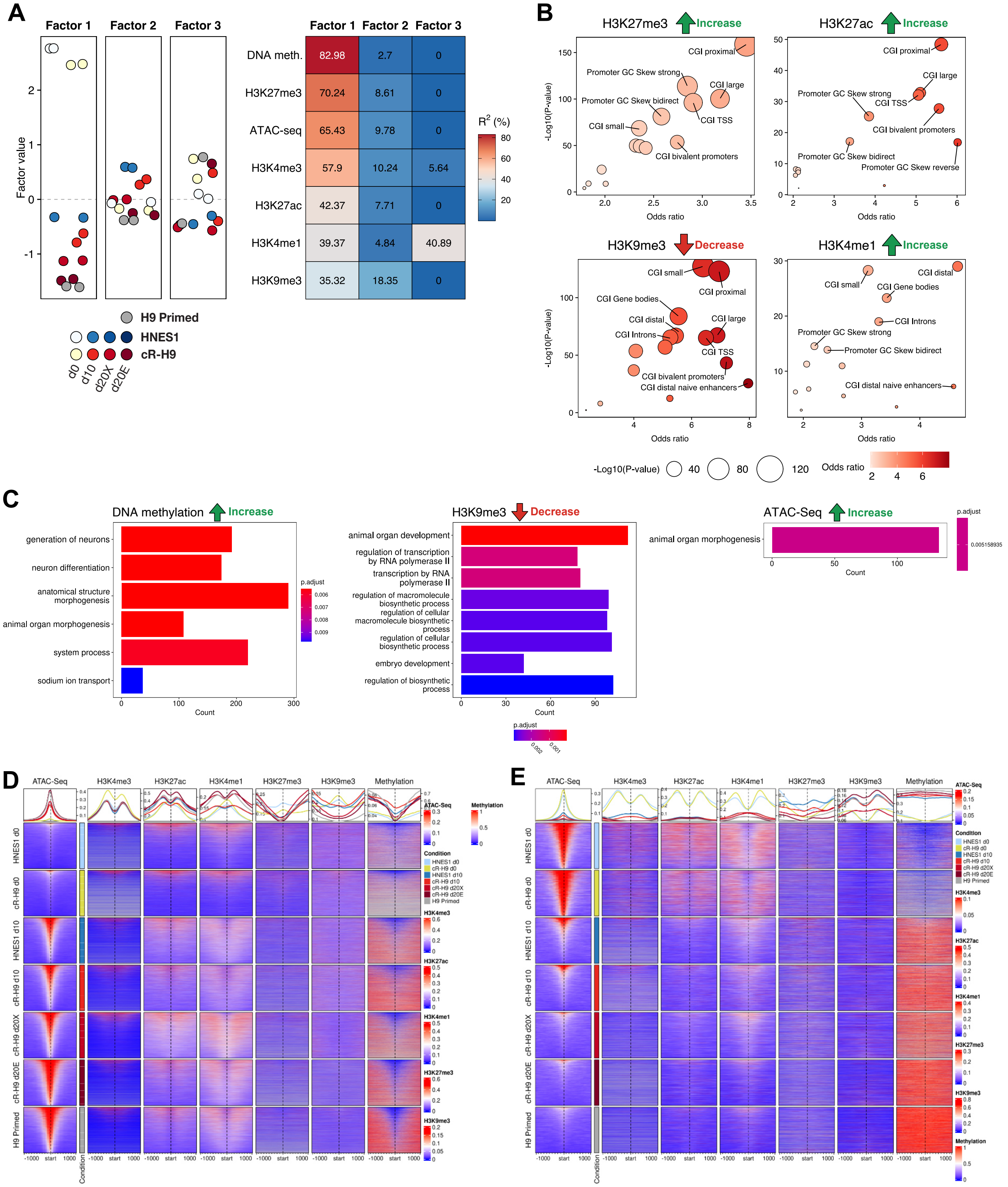

F

ATAC-seq Increase

Name	Matrix ID	E-value	Consensus	Width	Sites	E-value source	Motif source
**Pou5f1::Sox2**	MA0142.1	5.50E-54	ATTWGCATDWSAAW	14	2190	MEME	MEME
**Pou5f1::Sox2**	MA0142.1	5.70E-16	CWTTGTYATGCAAAT	15	5176	CENTRIMO	JASPAR2022
**TEAD4**	MA0809.2	9.80E-08	NNACATTCCARN	12	5799	CENTRIMO	JASPAR2022
**SOX10**	MA0442.2	2.50E-06	NDAACAAAGVN	11	5881	CENTRIMO	JASPAR2022
**Sox3**	MA0514.2	4.00E-06	DNACAATGGNN	11	5836	CENTRIMO	JASPAR2022
**SOX4**	MA0867.2	7.40E-06	RAACAAAGRV	10	5539	CENTRIMO	JASPAR2022
**SOX15**	MA1152.1	1.20E-05	CYWTTGTTHW	10	5942	CENTRIMO	JASPAR2022
**Sox5**	MA0087.2	1.50E-05	VRVAACAATGGNN	13	5434	CENTRIMO	JASPAR2022
**POU3F1**	MA0786.1	2.40E-05	WTATGCWAATKW	12	5199	CENTRIMO	JASPAR2022
**SOX2**	MA0143.4	4.10E-05	DNACAATGGNN	11	5874	CENTRIMO	JASPAR2022

ATAC-seq Decrease

Name	Matrix ID	E-value	Consensus	Width	Sites	E-value source	Motif source
		1.10E-10	TTAAGGG	7	669	MEME	MEME
**TFAP2B**	MA0811.1	1.60E-10	YGCCCBVRGGCA	12	2045	CENTRIMO	JASPAR2022
		2.00E-10	CTYYCTAATC	10	128	MEME	MEME
**TFAP2A**	MA0810.1	1.00E-09	YGCCCBVRGGCR	12	2092	CENTRIMO	JASPAR2022
**TFAP2C**	MA0524.2	6.00E-09	YGCCYBVRGGCA	12	2081	CENTRIMO	JASPAR2022
**RXRG**	MA0856.1	3.40E-08	GRGGTCAAAGGTCA	14	118	CENTRIMO	JASPAR2022
**MYBL1**	MA0776.1	7.50E-08	TTAAGGGY	8	284	STREME	STREME
		2.10E-07	ATGTGCTTTGT	11	184	STREME	STREME
**Sox3**	MA0514.2	5.70E-07	RRCAAADSAA	10	1180	MEME	MEME
**HMBOX1**	MA0895.1	1.10E-06	MYTAGTTAMS	10	2035	CENTRIMO	JASPAR2022

Fig. S8. Multimodal dynamics of epigenetic marks.
(**A**) MOFA2 factor values for the three factors extracted by analysing the differential peaks of histone modifications' ChIP-Seq, ATAC-Seq, and the top variable 200 CpG-containing regions based on methylation percentage between. On the right side, the variance explained (R-squared values) by each sequencing assay for the clustering samples in each MOFA2 factor. (**B**) Enrichment of factor 1 top loading weight features with CpG island and GC skew-associated genomic regions. Only the factor 1 features with significant overlap with CpG islands were selected. (**C**) Gene ontology of the closest genes (less than 50 kb) to MOFA2 factor 1 top loading weight features. Only the factor 1 features that returned gene ontology terms are represented. (**D**) Epigenetic profile and heatmap of regions centred at the ATAC-Seq peaks with high normalized counts in the primed state, selected by loading weight in factor 1. (**E**) Epigenetic profile and heatmap of regions centred at the ATAC-Seq peaks with high normalized counts in the naïve state, selected by loading weight in factor 1. (**F**) Top results of the MEME Suite motif analysis conducted on the ATAC-Seq peaks selected based on the highest factor 1 loading weight features. On the left side, the motif results for the regions that show an increased accessibility from naïve to primed and, on the right side, the motif results for the regions that show a decreased accessibility from naïve to primed.

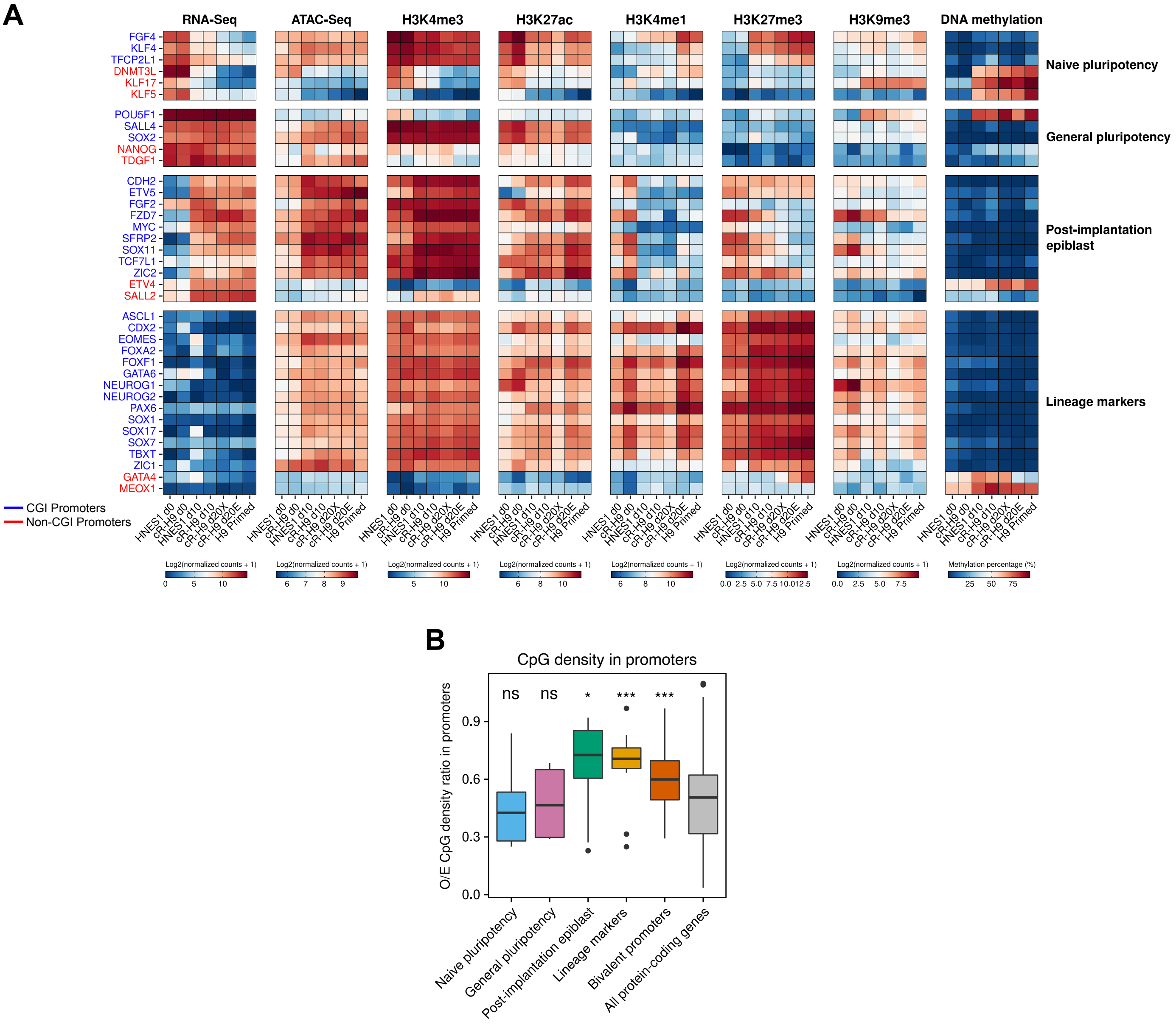

Fig. S9. Association between promoter epigenetic state and gene expression in selected genes. (**A**) Gene expression and promoter ATAC-Seq and ChIP-Seq log-transformed normalized counts, and methylation percentage for genes associated with naïve pluripotency, general pluripotency, post-implantation epiblast expression, and lineage expression in each condition. (**B**) Promoter CpG observed/expected density ratio comparison for genes associated with naïve pluripotency, general pluripotency, post-implantation epiblast expression, and lineage expression, genes with bivalent promoters in all conditions, and all protein-coding genes. Statistical test comparison against all protein-coding genes. ns - non-significant, *P < 0.05, **P < 0.01, and ***P < 0.001; Student's t-test.

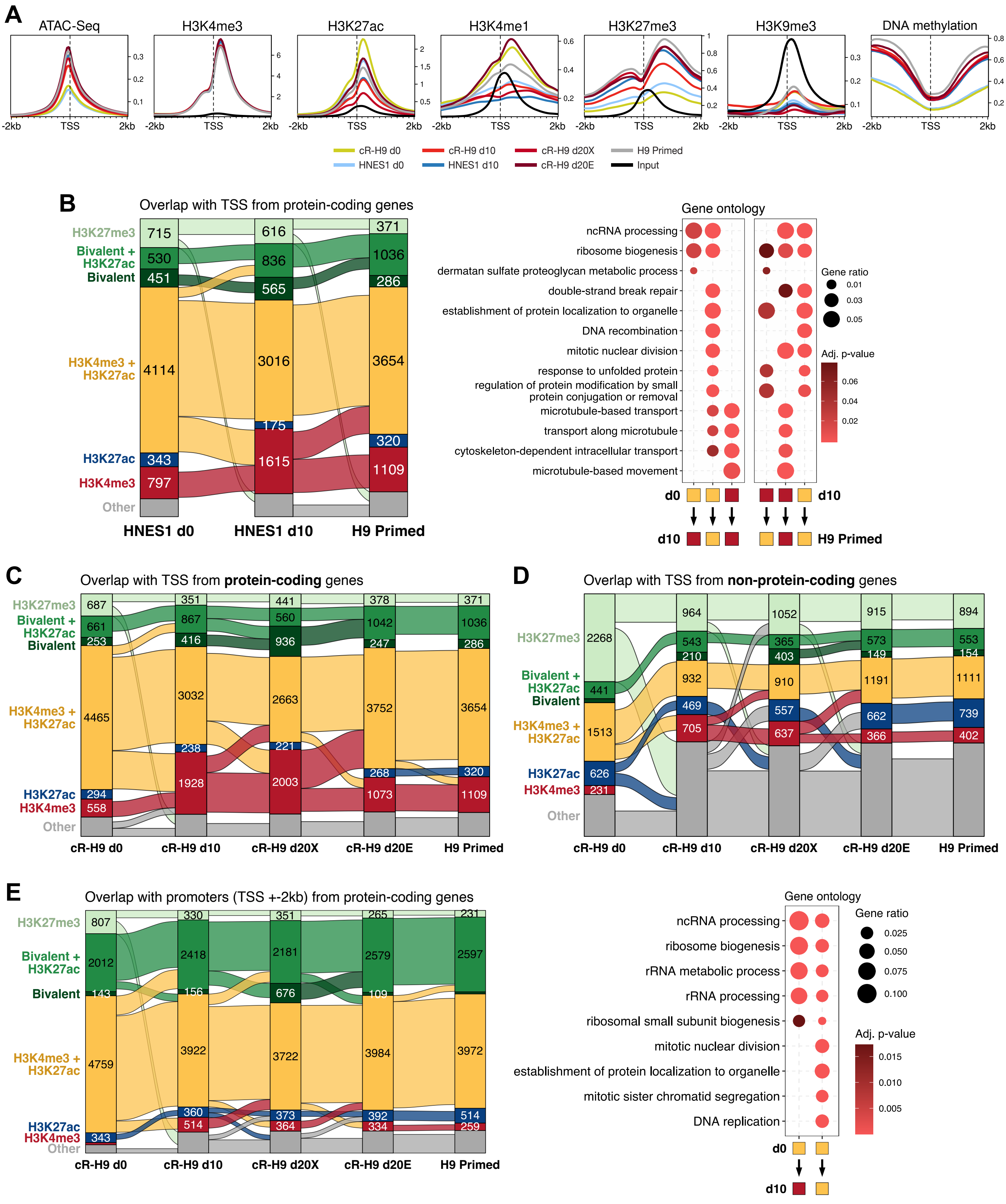
A
ATAC-Seq
H3K4me3
H3K27ac
H3K4me1
H3K27me3
H3K9me3
DNA methylation
-2kb
TSS
2kb
cR-H9 d0
HNES1 d0
cR-H9 d10
HNES1 d10
cR-H9 d20X
cR-H9 d20E
H9 Primed
Input
B
Overlap with TSS from protein-coding genes
H3K27me3
Bivalent + H3K27ac
Bivalent
H3K4me3 + H3K27ac
H3K27ac
H3K4me3
Other
715
530
451
4114
343
797
616
836
565
3016
175
1615
371
1036
286
3654
320
1109
HNES1 d0
HNES1 d10
H9 Primed
Gene ontology
ncRNA processing
ribosome biogenesis
dermatan sulfate proteoglycan metabolic process
double-strand break repair
establishment of protein localization to organelle
DNA recombination
mitotic nuclear division
response to unfolded protein
regulation of protein modification by small protein conjugation or removal
microtubule-based transport
transport along microtubule
cytoskeleton-dependent intracellular transport
microtubule-based movement
Gene ratio
0.01
0.03
0.05
Adj. p-value
0.06
0.04
0.02
d0
d10
d10
H9 Primed
C
Overlap with TSS from **protein-coding** genes
H3K27me3
Bivalent + H3K27ac
Bivalent
H3K4me3 + H3K27ac
H3K27ac
H3K4me3
Other
687
661
253
4465
294
558
351
867
416
3032
238
1928
441
560
936
2663
221
2003
378
1042
247
3752
268
1073
371
1036
286
3654
320
1109
cR-H9 d0
cR-H9 d10
cR-H9 d20X
cR-H9 d20E
H9 Primed
D
Overlap with TSS from **non-protein-coding** genes
H3K27me3
Bivalent + H3K27ac
Bivalent
H3K4me3 + H3K27ac
H3K27ac
H3K4me3
Other
2268
441
1513
626
231
964
543
210
932
469
705
1052
365
403
910
557
637
915
573
149
1191
662
366
894
553
154
1111
739
402
cR-H9 d0
cR-H9 d10
cR-H9 d20X
cR-H9 d20E
H9 Primed
E
Overlap with promoters (TSS +-2kb) from protein-coding genes
H3K27me3
Bivalent + H3K27ac
Bivalent
H3K4me3 + H3K27ac
H3K27ac
H3K4me3
Other
807
2012
143
4759
343
330
2418
156
3922
360
514
351
2181
676
3722
373
364
265
2579
109
3984
392
334
231
2597
3972
514
259
cR-H9 d0
cR-H9 d10
cR-H9 d20X
cR-H9 d20E
H9 Primed
Gene ontology
ncRNA processing
ribosome biogenesis
rRNA metabolic process
rRNA processing
ribosomal small subunit biogenesis
mitotic nuclear division
establishment of protein localization to organelle
mitotic sister chromatid segregation
DNA replication
Gene ratio
0.025
0.050
0.075
0.100
Adj. p-value
0.015
0.010
0.005
d0
d10

Fig. S10. Promoter classification dynamics based on overlap with histone modifications
(**A**) Read profiles from ATAC-Seq, H3K4me3, H3K27ac, H3K4me1, H3K27me3, H3K9me3 histone modifications, and methylation percentage overlapping the TSS (transcription start site) plus and minus 2 kilobases in all protein-coding genes. (**B**) Number of protein-coding genes with their TSS categorized based on having an overlap with the following combination of ChIP-Seq peaks in HNES1 and H9 Primed cells: only H3K27me3, 'H3K27me3'; H3K4me3 with H3K27me3 and H3K27ac, 'Bivalent + H3K27ac'; H3K4me3 with H3K27me3, 'Bivalent'; H3K4me3 with H3K27ac, 'H3K4me3 + H3K27ac'; only H3K27ac, 'H3K27ac'; only H3K4me3, 'H3K4me3'; and the category 'Other' containing all the remaining combinations between the epigenetic marks H3K4me3, H3K27ac, and H3K27me3. On the right side, the gene ontology from selected category transitions between conditions. (**C**) Number of protein-coding genes with their TSS categorized based on having an overlap with the combinations of H3K4me3, H3K27ac, and H3K27me3 peaks in all H9 cell conditions. (**D**) Number of non-protein-coding genes with their TSS categorized based on having an overlap with the combinations of H3K4me3, H3K27ac, and H3K27me3 peaks in all H9 cell conditions. (**E**) Number of protein-coding genes with their promoter (TSS +-2 kb) categorized based on having an overlap with the combinations of H3K4me3, H3K27ac, and H3K27me3 peaks in all H9 cell conditions. On the right side, the gene ontology from selected category transitions between conditions. All results in this figure were limited to autosomes.

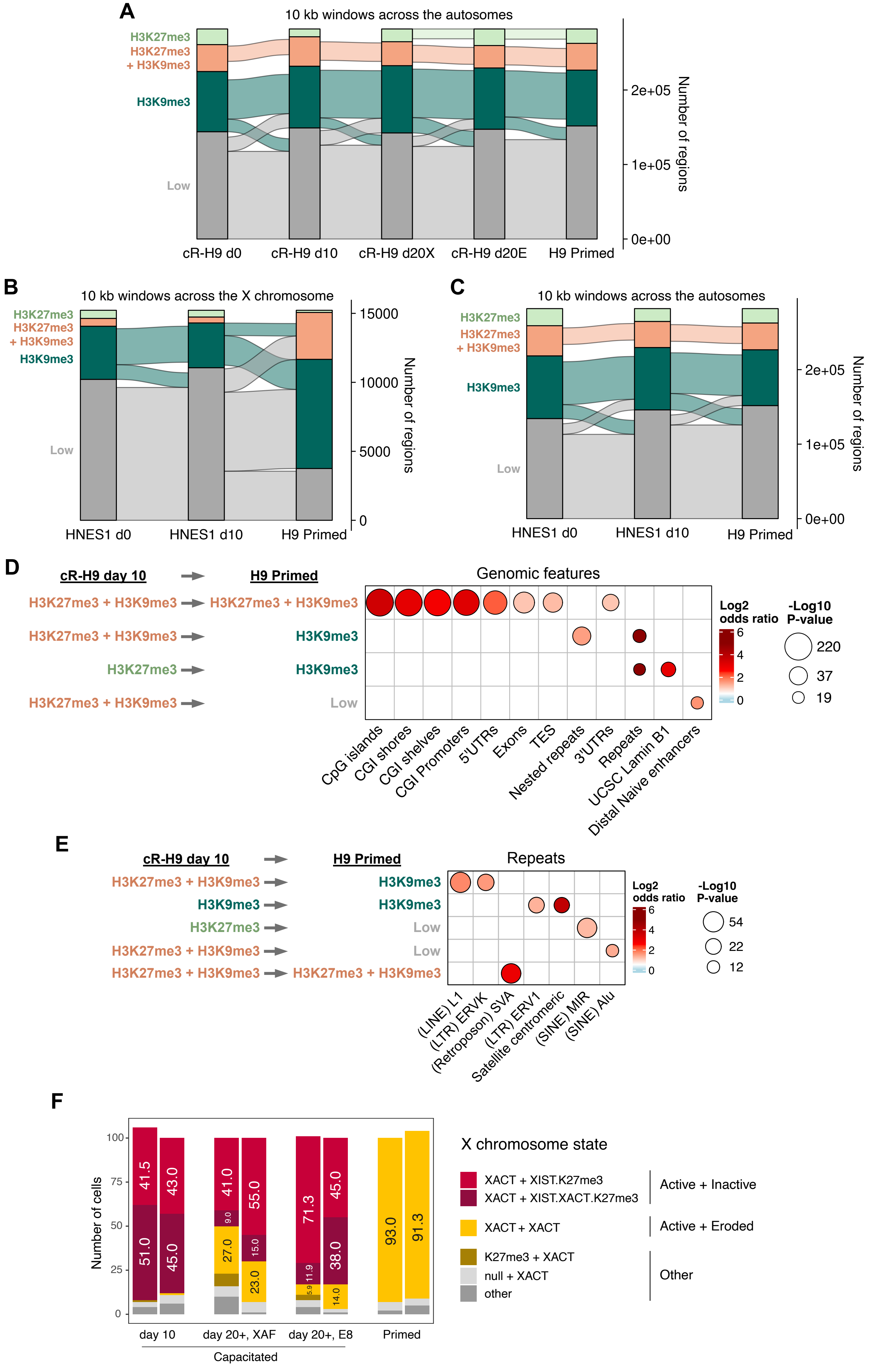

A
10 kb windows across the autosomes
H3K27me3
H3K27me3
+ H3K9me3
H3K9me3
Low
cR-H9 d0
cR-H9 d10
cR-H9 d20X
cR-H9 d20E
H9 Primed
Number of regions
0e+00
1e+05
2e+05
B
10 kb windows across the X chromosome
H3K27me3
H3K27me3
+ H3K9me3
H3K9me3
Low
HNES1 d0
HNES1 d10
H9 Primed
Number of regions
0
5000
10000
15000
C
10 kb windows across the autosomes
H3K27me3
H3K27me3
+ H3K9me3
H3K9me3
Low
HNES1 d0
HNES1 d10
H9 Primed
Number of regions
0e+00
1e+05
2e+05
D
cR-H9 day 10
H9 Primed
H3K27me3 + H3K9me3
H3K27me3 + H3K9me3
H3K27me3 + H3K9me3
H3K9me3
H3K27me3
H3K9me3
H3K27me3 + H3K9me3
Low
Genomic features
CpG islands
CGI shores
CGI shelves
CGI Promoters
5'UTRs
Exons
TES
Nested repeats
3'UTRs
Repeats
UCSC Lamin B1
Distal Naive enhancers
Log2 odds ratio
6
4
2
0
-Log10 P-value
220
37
19
E
cR-H9 day 10
H9 Primed
H3K27me3 + H3K9me3
H3K9me3
H3K9me3
H3K9me3
H3K27me3
Low
H3K27me3 + H3K9me3
Low
H3K27me3 + H3K9me3
H3K27me3 + H3K9me3
Repeats
(LINE) L1
(LTR) ERVK
(Retroposon) SVA
(LTR) ERV1
Satellite centromeric
(SINE) MIR
(SINE) Alu
Log2 odds ratio
6
4
2
0
-Log10 P-value
54
22
12
F
Number of cells
0
25
50
75
100
41.5
51.0
43.0
45.0
41.0
9.0
27.0
55.0
15.0
23.0
71.3
11.9
5.9
45.0
38.0
14.0
93.0
91.3
day 10
day 20+, XAF
day 20+, E8
Primed
Capacitated
X chromosome state
XACT + XIST.K27me3
XACT + XIST.XACT.K27me3
Active + Inactive
XACT + XACT
Active + Eroded
K27me3 + XACT
null + XACT
other
Other

Fig. S11. X chromosome epigenetic states in female hPSCs after resetting and capacitation.
(**A**) Flow chart illustrating the number of 10-kilobase windows in autosomes classified based on H3K27me3 and H3K9me3 scaled (between 0 to 1) log2 normalized counts threshold of 0.5. The classification includes windows containing high levels of H3K27me3 marks ('H3K27me3'), high levels of H3K9me3 marks ('H3K9me3'), high levels of both H3K27me3 and H3K9me3 marks ('H3K27me3 + H3K9me3'), and low levels of both marks ('Low'). The chart shows the results for H9 cells only. (**B**) Flow chart illustrating the number of 10-kilobase windows in the X chromosome of HNES1 and H9 Primed cells classified based on H3K27me3 and H3K9me3 scaled (between 0 to 1) log2 normalized counts threshold of 0.5. (**C**) Flow chart illustrating the number of 10-kilobase windows in the autosomes of HNES1 and H9 Primed cells classified based on H3K27me3 and H3K9me3 scaled (between 0 to 1) log2 normalized counts threshold of 0.5. (**D**) Enrichment of regions with classification transition between cR-H9 day 10 and H9 Primed cells over annotated genomic features. Only results with an FDR (false discovery rate) below 0.05, absolute odds ratio value above 2, and a negative log p-value above 10 are shown. (**E**) Enrichment of regions with classification transition between cR-H9 day 10 and H9 Primed cells over annotated repetitive families. Only results with an FDR below 0.05, absolute odds ratio value above 2, and a negative log p-value above 10 are shown. (**F**) Detailed breakdown of cell categories identified using the immuno-FISH shown in Fig. 6H.

Tables

Table S1. Normalised gene expression matrix obtained by integrating single-cell RNA-Seq datasets of human and non-human primate embryonic epiblast (*25–27*).

Table S2. Normalised gene expression matrix from hPSCs during capacitation (*9*).

Table S3. Lists of the most variable genes during the embryonic epiblast development and *in vitro* capacitation.

Table S4. Gene ontology terms enrichment analysis related to Fig. 1.

Table S5. Overview of the number of biological replicates used in each condition and library strategy. The condition categories include '0' (0 days), '1' (1 day), '2' (2 days), '3' (3 days), '7' (7 days), '10' (10 days), '20X' (more than 10 days in XAF medium after the 10 days in the XAV medium), '20E' (more than 10 days in E8 medium after the 10 days in the XAV medium), and 'Conventional Primed control' (H9 cells cultured in E8 medium).

Table S6. Results from the LOLA enrichment analysis applied to each state of the ChromHMM 13-state model. Only results with an odds ratio above 1 and a negative log p-value above 2 are included.

Table S7. List of human allele-specific differentially methylated regions (DMRs), indicating known, novel, and placental-specific imprinted regions. Each column provides the following information: 'Status' describes the detected DMRs; 'Gene' provides the DMR name attributed by matching the name of the nearest gene; 'Chr' indicates the chromosome number; 'Start' and 'End' give the chromosomal start and end positions, respectively; 'Inf' shows the number of informative alleles; 'CG content' provides the ratio of the number of cytosines (C) and guanines (G) over the total number of nucleotides; '#CpG' indicates the number of CpGs present within the DMR; and 'Origin' specifies the parental allele where the imprinting is observed, with 'M' indicating maternal and 'P' indicating paternal.

Table S8. List of genes from which their corresponding protein products that belong to the Polycomb-group proteins (PcG): PRC1, PRC1, and PR-DUB complex; Trithorax-group proteins (TrxG): COMPASS, MLL1/MLL2, MLL3/MLL4, SET1, and SWI/SNF complex; the DNMT, TET, and IDH protein families; and histone acetyltransferases, histone deacetylases, and histone demethylates. The table contains the following information: 'Gene name' gives the official gene symbol and 'Protein group' indicates the protein family to which the protein product belongs to. Additionally, the table provides the average RNA-Seq log normalized counts for the HNES1 and cR-H9 cells in days 0, 1, 2, 3, 7, 10, '20X' (more than 10 days in XAF medium after the 10 days in the XAV medium), '20E' (more than 10 days in E8 medium after the 10 days in the XAV medium), and the RNA-Seq log normalized counts for the H9 cells cultured in E8 medium.

Table S9. Results from the differential expression of transposable elements analysis. The column 'comparison' indicates the differential comparison between conditions. The results are divided into our RNA-Seq dataset, Zijlmans et al. (2022) (*19*) dataset, Kumar et al. (2022) (*31*) dataset, and Collinson et al. (2016) (*30*) dataset.

Table S10. Results from the motif enrichment analysis performed using the software MEME-ChIP from the MEME Suite. The results are divided into ATAC-Seq regions that had a significant read count decrease between naïve and primed, 'ATAC-Seq Naïve', and ATAC-Seq regions that had a significant read count increase between naïve and primed, 'ATAC-Seq Primed'.

Table S11. List of genes belonging to the RNA-Seq clusters and their average RNA-Seq log normalized counts of the HNES1 and cR-H9 cells in days 0, 1, 2, 3, 7, 10, '20X' (more than 10 days in XAF medium after the 10 days in the XAV medium), '20E' (more than 10 days in E8 medium after the 10 days in the XAV medium), and the RNA-Seq log normalized counts for the H9 cells cultured in E8 medium.

Table S12. Gene ontology terms that were significantly enriched from genes belonging to the RNA sequencing clusters. Each row represents a different gene ontology term. Only gene ontology terms with a significant enrichment (adjusted p-value < 0.1) are included in the table.

Table S13. List of genes classified by their association with naïve pluripotency, primed pluripotency, post-implantation epiblast, and lineage-specific expression, obtained from Rostovskaya et al., 2019 (*9*).

Table S14. Gene ontology terms that were significantly enriched from the protein-coding genes that had their transcription start site (TSS) was categorized based on having an overlap with the epigenetic marks H3K4me3, H3K27ac, and H3K27me3 and then clustered based on their TSS classification transition between day 0 to day 10 and day 10 to H9 Primed. The results are separated into the cR-H9 and HNES1 cells. Each row represents a different gene ontology term. Only gene ontology terms with a significant enrichment (adjusted p-value < 0.1) are included in the table.

Table S15. List of software packages and databases used during data processing and analysis.

Table S16. List of publicly available sequencing datasets used in the study.

Table S17. List of antibodies.

Table S18. List of qPCR primers.
